# Filling the Gaps in the Kirromycin Biosynthesis: Deciphering the Role of Genes Involved in Ethylmalonyl-CoA Supply and Tailoring Reactions

**DOI:** 10.1038/s41598-018-21507-6

**Published:** 2018-02-19

**Authors:** Helene L. Robertsen, Ewa M. Musiol-Kroll, Ling Ding, Kristina J. Laiple, Torben Hofeditz, Wolfgang Wohlleben, Sang Yup Lee, Stephanie Grond, Tilmann Weber

**Affiliations:** 1 Novo Nordisk Foundation Center for Biosustainability, Technical University of Denmark, Kemitorvet building 220, 2800 Kgs. Lyngby, Denmark; 2 Eberhard-Karls-Universität Tübingen, Interfakultäres Institut für Mikrobiologie und Infektionsmedizin, Mikrobiologie / Biotechnologie, Auf der Morgenstelle 28, 72076 Tübingen, Germany; 3 German Centre for Infection Research (DZIF), Partner site Tübingen, Auf der Morgenstelle 28, 72076 Tübingen, Germany; 4 Eberhard-Karls-Universität Tübingen, Institut für Organische Chemie, Auf der Morgenstelle 18, 72076 Tübingen, Germany; 5 Department of Chemical and Biomolecular Engineering (BK21 Plus Program), Korea Advanced Institute of Science and Technology (KAIST), 291 Daehak-ro, Yuseong-gu, Daejeon, 305-701 Republic of Korea

## Abstract

Kirromycin is the main product of the soil-dwelling *Streptomyces collinus* Tü 365. The elucidation of the biosynthetic pathway revealed that the antibiotic is synthesised via a unique combination of *trans*-/*cis*-AT type I polyketide synthases and non-ribosomal peptide synthetases (PKS I/NRPS). This was the first example of an assembly line integrating the three biosynthetic principles in one pathway. However, information about other enzymes involved in kirromycin biosynthesis remained scarce. In this study, genes encoding tailoring enzymes KirM, KirHVI, KirOI, and KirOII, and the putative crotonyl-CoA reductase/carboxylase KirN were deleted, complemented, and the emerged products analysed by HPLC-HRMS and MS/MS. Derivatives were identified in mutants Δ*kirM*, Δ*kirHVI*, Δ*kirOI*, and Δ*kirOII*. The products of Δ*kirOI*, Δ*kirOII*, and *kirHVI* were subjected to 2D-NMR for structure elucidation. Our results enabled functional assignment of those enzymes, demonstrating their involvement in kirromycin tailoring. In the Δ*kirN* mutant, the production of kirromycin was significantly decreased. The obtained data enabled us to clarify the putative roles of the studied enzymes, ultimately allowing us to fill many of the missing gaps in the biosynthesis of the complex antibiotic. Furthermore, this collection of mutants can serve as a toolbox for generation of new kirromycins.

## Introduction

Streptomycetes are known for their profound ability to produce a diverse set of secondary metabolites with relevant pharmaceutical properties, including, but not limited to, antibiotics, anti-fungals, immunosuppressant, and antitumor agents. Polyketides comprise a noteworthy subset of these metabolites. They played and still play an important role in the clinic^1,2^, hence highlighting the importance of their discovery.

**Figure 1:**
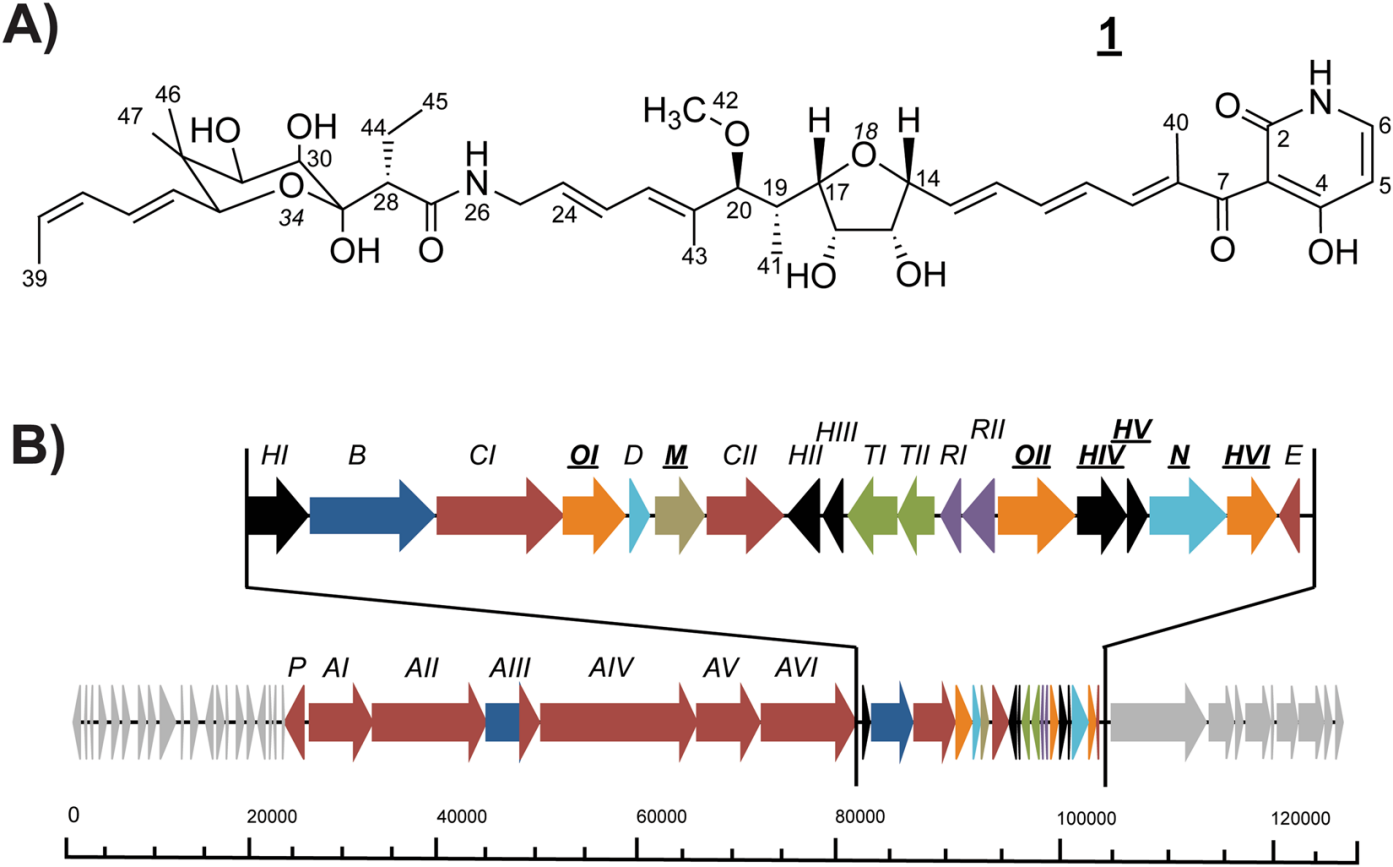
The structure and biosynthetic gene cluster of kirromycin. (**A**) Kirromycin is composed of three intramolecular ring structures; a pyridone ring, a central tetrahydrofuran (THF) ring, and a sugar-like moiety termed goldinonic acid^4^. (**B**) Modified graphics from Weber *et al*.^4^. Black, Hypothetical proteins; Blue, NRPS-related genes; Red, PKS-related genes; Orange, Dehydrogenases and hydroxylases; Light blue, Genes involved in precursor supply; Light brown, O-methyltransferase; Light green, Transport-related genes; Purple, Regulatory genes; Light grey, Genes putatively not involved in kirromycin biosynthesis. Genes studied here are in bold and have been underlined.

Polyketides are synthesised by megaenzyme complexes termed modular polyketide synthases (PKSs), which catalyse the controlled initiation and elongation of simple carboxylic acid monomers into polyketide chains. One example of a complex polyketide is the narrow-spectrum antibiotic kirromycin. The compound was first isolated from the soil-dwelling *Streptomyces collinus* Tü 365 in 1972^3^. The linear molecule is composed of three internal ring structures: a pyridone ring, a central tetrahydrofuran (THF) group, and a sugar-like moiety, also referred as goldinonic acid^4^ (Fig. 1A). These three kirromycin moieties are directly involved in the binding of the antibiotic kirromycin to the elongation factor (EF) Tu in prokaryotes^5,6^. This interaction prevents the conformational change between the GTP- and GDP-bound form of the EF Tu, which results in stalling of protein biosynthesis.

Although the structure and activity of kirromycin were known since the early 70s, most of the biosynthetic steps remained unexplored. After the 82 kb kirromycin cluster (Fig. 1B), encoding 28 genes, was identified and described^4^, more details on the assembly of the compound were obtained. The hypothetical pathway of kirromycin indicated that the biosynthetic steps involve non-ribosomal peptide synthetases (NRPSs), acyltransferase (AT)-harbouring PKSs (*cis*-AT PKSs), AT-less PKSs (*trans*-AT PKSs), and several tailoring enzymes. This first description of a biosynthetic pathway involving three different types of multi-modular enzymes has made the assembly line a relevant model for studying complex *cis*-/*trans*-AT PKS/NRPS pathways.

Directly upstream of the PKS/NRPS genes in the BGC of kirromycin, the Sfp-type phosphopantetheinyl transferase (PPTase) KirP, responsible for the post-translational activation of PKS acyl carrier proteins (ACPs) and NRPS peptidyl carrier proteins (PCPs), was identified^7^. The core PKS/NRPS enzymes in kirromycin are encoded by *kirAI – kirAVI* and *kirB*^4^. Closer examinations of the PKS-related genes and their products revealed KirAVI to be the only *cis*-AT PKS in the assembly line. The upstream PKSs, KirAI – KirAV, lack the integrated ATs. For these modules, extender units are provided by the discrete ATs KirCI^8,9^ and KirCII^8,10^, which provide malonyl-CoA and ethylmalonyl-CoA to the ACPs of the assembly line. The last core enzyme of the kirromycin PKS/NRPS assembly line is encoded by *kirB*. The protein KirB is responsible for incorporation of the precursor β-alanine^4^, which is biosynthesised by the aspartate-α-decarboxylase encoded by *kirD*^11^. Upon release from the assembly line, this precursor is cyclised to form the pyridone ring^4^. Interestingly, no genes encoding a typical thioesterase (TE) were found in the kirromycin biosynthetic gene cluster (BGC). Recently, Gui *et al*.^12^ reported the discovery of a new group of Dieckmann cyclases involved in the biosynthesis of tetramic acid and pyridone scaffolds. A member of this new family of enzymes was also identified in the kirromycin BGC. KirHI, previously assigned with a hypothetical function, was found to catalyse pyridone ring closure and the co-occurring release of the molecule chain from the PKS I/NRPS assembly line^12^.

While the precursor loading of the PKS I/NRPS modules and the kirromycin polyketide chain elongation are well investigated, only limited knowledge about the provision of ethylmalonyl-CoA and the final tailoring reactions is available for this pathway. To date, the underlying reactions leading to the THF ring closure, the introduction of hydroxyl groups at C-16 and C-30, methylation of the oxygen at O-42, and the formation of the double bond between C-5 and C-6 in the pyridone ring have remained elusive. These enzymatic reactions cannot be explained from the enzymatic domains encoded within the PKS I/NRPS complex, and thus it was speculated that those product modifications are achieved through tailoring reactions.

In this study, the genes *kirM*, *kirHVI*, *kirOI*, *kirOII*, *kirHIV*, *kirHV*, and *kirN*, which are encoded in the kirromycin BGC but have no experimentally determined function, were examined by genetic inactivation and gene complementation. The compounds produced by the gene inactivation mutants were analysed and characterised by high performance liquid chromatography (HPLC)–high-resolution mass spectrometry (HRMS), and MS/MS. In addition, nuclear magnetic resonance (NMR) was employed for structural elucidation of the derivative produced by the Δ*kirHVI*, Δ*kirOI*, and Δ*kirOII* mutants.

## Methods

### Bacterial strains and general growth conditions

Plasmids and strains used in this study are listed in Supplementary Table S1. Wild type strain *Streptomyces collinus* Tü 365 was obtained from the “Tübinger Stammsammlung”. For routine cultivations of mutants, complemented mutants, and wild type strain, Tryptic Soy Broth (TSB) supplemented with nalidixic acid (25 μg/mL) was used. Furthermore, complemented mutants were propagated in the presence of apramycin (50 μg/mL). Sporulation of wild type and mutant strains was performed on modified SFM agar (2% mannitol, 2% full fat soy flour, 20 mM MgCl_2_, 20 mM CaCl_2_, tap water). *Escherichia coli* DH5-α was used for standard cloning procedure of pGUSA21, pDrive, pA18mob, pJet1.2/blunt, pGM1190, and pRM4 constructs. Transformation was carried out according to the manufacturer’s specifications and including a 1 h recovery step in Super Optimal broth with Catabolite repression (S.O.C.) medium. *E*. *coli* was grown at 37 °C (200 rpm) in Lysogeny broth (LB) medium supplemented with appropriate antibiotics. In order to introduce the construct into streptomycetes, plasmid DNA was introduced into *E*. *coli* ET12567 (pUZ8002) by calcium chloride transformation. ET12567 strains were grown in LB supplemented with apramycin (50 μg/mL), chloramphenicol (25 μg/mL), and kanamycin (25 μg/mL) for 16 hours, shaking at 200 rpm at 37 °C.

### Construction of *kirM*, *kirN*, *kirHIV*, and *kirHV* gene inactivation plasmids

All primers used in this study are listed in Supplementary Table S2. A 2.1 kb cassette, containing the ermE* promoter flanked by two 1 kb-fragments up- and downstream of the genes *kirM*, *kirHIV*, *kirHV*, and *kirN*, were amplified from pCRISPR-Cas9_USER- *kirM/kirHIV/kirHV/kirN*, which were constructed based on a previously described method^13^ (see Supplementary Table S1). For the amplifications, primers KP1 – KP8 and PCR programs listed in Supplementary Table S2 were used. Each of the *kir* cassettes were assembled in pGUSA21^14^ by Gibson cloning carried out at 50 °C for 30 min. Correct clones of pHR1 and pHR3–5 were identified by control PCR and confirmed by Sanger sequencing with KP37/KP42.

### Construction of *kirHVI* gene inactivation plasmid

To generate the *kirHVI* gene inactivation plasmid pHR2, 1 kb fragments, flanking the target gene, were amplified from the cosmid 2K05 DNA^4^ using primers KP9/KP10 and KP13/KP14. The ermE* promoter was amplified from plasmid pCRISPR-Cas9^15^ with primers KP11/KP12. 1 M betaine was added to the PCRs to improve amplification of GC-rich stretches. Upon gel purification, the three fragments were assembled in pGUSA21^14^ by Gibson cloning carried out at 50 °C for 30 min, yielding pHR2. Correct clones of pHR2 were identified by control PCR and Sanger sequencing with KP37/KP38.

### Construction of *kirOI* and *kirOII* gene inactivation plasmids

To construct pTL-*kirOI*, 1.2 kb of the flanking regions of the gene were amplified by PCR with primer pairs KP15/KP16 and KP17/KP18 using the PCR programs listed in Supplementary Table S2. For construction of pDW-*kirOII*, 2 kb of the flanking regions of *kirOII* were amplified by PCR with primers KP21/KP22 and KP23/KP24 using the PCR program listed in the Supplementary Table S2.

The left and right fragments, up- and downstream of *kirOI* and *kirOII*, were cloned into pA18mob resulting in the plasmids pTL-*kirOI* and pDW-*kirOII*, respectively. The thiostrepton resistance cassette (1.1 kb) was amplified from plasmid pSLE61^16^ with primers KP19/KP20. The 1.1 kb fragment was first cloned into pDrive (Qiagen, Hilden, Germany) and then inserted via the XbaI sites into pTL-*kirOI* and pDW-*kirOII*, resulting in the plasmids pTL-*kirOI*-thio and pDW-*kirOII*-thio, respectively. The gene replacement mutants were confirmed by control PCRs targeting the apramycin and thiostrepton resistance cassettes with the primer pairs KP39/KP40 and KP19/KP20, respectively.

To distinguish between wild type and single/double crossover mutants, the primer pairs KP41/KP42 and KP43/KP44 were used for amplification of an internal region of (wild type) *kirOI/kirOII* or the thiostrepton resistance marker (single- or double crossover mutant).

In addition to PCR, Southern Blot analysis was used to confirm correct mutants of *kirOI* and *kirOII*. Here, the DIG-labelled probe for Southern Blot experiments was amplified from cosmid 1C24 DNA^4^ with the primer pairs KP41/KP42 (*kirOI*) or KP43/KP44 (*kirOII*) using a 10 × DIG DNA labelling mix (Roche, Mannheim, Germany). Genomic DNA preparation from streptomycetes was performed with the NucleoSpin® Tissue Kit from Macherey-Nagel, Düren, Germany.

### Intergeneric conjugation in *S*. *collinus* Tü 365

To introduce the gene inactivation plasmids into *S*. *collinus* Tü 365, a standard protocol for intergeneric conjugation was used^17^. Single crossover mutants were selected based on blue-white screening^14^ in which spore dilution plates were overlaid with 20 mM X-Gluc (5-bromo-4-chloro-3-indolyl-β-D-glucuronic acid). Control PCR was used to verify the single crossovers using primers KP72/KP73. For induction of the double crossover event, two-day-old cultures of single crossover mutants, cultivated in TSB medium, were stressed for 24 h at 37 °C, shaking at 160 rpm. Spore dilution plates were overlaid with 20 mM X-Gluc and white clones were picked for control PCRs with primers KP45 – KP58 (see Supplementary Fig. S6).

### Construction of complementation plasmids for kirromycin *kir* mutants

*kir* genes were PCR amplified using the cosmid 2K05 (*kirM*, *kirHVI*, *kirOI*, *kirOII*, and *kirN*)^4^ as template and the primers KP27 – KP36. 1 M betaine was added to the PCRs to improve amplification of the GC-rich stretches. The DNA encoding the *kir* genes was purified from gel and cloned into the blunt end pJet1.2 vector as instructed by the manufacturer. Correct clones were verified by control PCR and Sanger sequencing with primers KP59/KP60.

pJet1.2 clones of *kirM*, *kirHVI*, and *kirN* were subjected to restriction digestions with NdeI and HindIII together with DNA of integrative plasmid pRM4^18^. The pJet1.2 clone of *kirOII* was subjected to restriction digestion with NheI and HindIII together with pRM4. Finally, pJet1.2-*kirOI* was subjected to restriction digestion with HindIII and EcoRI together with replicative plasmid pGM1190. The genes purified from gel and linearised vectors were ligated using T4 DNA Ligase and introduced into *E*. *coli* DH5-α competent cells according to manufacturer’s instructions. The complementation plasmids of pGM1190 and pRM4 were verified by control PCR and Sanger sequencing with primers KP61/KP62 and KP63/KP64, respectively, and transferred to *S*. *collinus* mutant strains by standard intergeneric conjugation^17^.

### Kirromycin production assay, HPLC-HRMS, and MS/MS analyses

For the fermentations, 5 mL of two days-old precultures (grown in TSB) were used for inoculation of 95 mL kirromycin production medium composed of 1% full-fat soy flour, 1% D-mannitol, and 0.5% CaCO_3_ dissolved in tap water and pH adjusted to 7.4 prior to autoclaving. Fermentations were carried out for six days at 30 °C in a rotary shaker at 160 rpm. Cultures were extracted with 1:1 ethyl acetate for 90 minutes and evaporated in a Büchi® Rotavapor® RII evaporator equipped with jack and water bath. Dried extracts were redissolved in 500 μL methanol. HPLC–HRMS analysis was carried out with an Orbitrap Fusion connected to a Dionex Ultimate 3000 UHPLC pumping system (ThermoFisher Scientific, Waltham, MA, USA). UV-Vis detection was done using a DAD-3000 set to the range 200–600 nm. Samples were kept at 10.0 °C in the autosampler during the analysis. 2 μL of each sample was injected into a C18 Acquity UPLC F5–3 HPLC column (2.1 × 100 mm, 1.8 μm,) at a flow rate of 0.4 *m*L/min, 30.0 °C. Mobile phases A and B were 0.1% formic acid in water and acetonitrile, respectively. Elution was done with a 30 min multistep system. After 5% B for 1 min, a linear gradient started from 5% B to 100% B in 21 min, which was held for another 5 min and followed by re-equilibration to 5% B until 30 min. Data was collected in both positive and negative ion modes with a scan range of (m/z) = 200–2000. MS/MS fragmentation was carried out using Ion Trap for collision-induced dissociation (CID) with the collision energy (30%), RF Lens 60%, AGC target 5.0e4, and scan range (m/z) 230–800. Data analyses were performed with the software Xcalibur 3.0.63 (Thermo Fisher Scientific Inc.).

### NMR of 30-deoxy-kirromycin, 5,6-dihydro-kirromycin, and 30-hydroxy-5,6-dehydro-1-*N*-demethyl-16-deoxy-kirrothricin

To confirm the structure of the 30-hydroxy-5,6-dehydro-1-*N*-demethyl-16-deoxy-kirrothricin (kirromycin-Δ*kirOII*, **5**), a 2L-fermentation of the *ΔkirOII* mutant was carried out using the same production medium and extraction conditions described previously. Pure compound **5** (2.2 mg) was obtained by separation on a semi-preparative RP-18 column by HPLC. 1D and 2D NMR were acquired using standard pulse sequences on an 800 MHz Bruker Avance spectrometer with a 5 mm TCI cryo probe at NMR Center, Technical University of Denmark. CD_3_OD was applied as solvent. Chemical shifts were usually expressed in parts per million (ppm, *δ*) relative to internal standard tetramethylsilane (TMS).

In addition, 30-deoxy-kirromycin (kirromycin-Δ*kirHVI*, **3**), 5,6-dihydro-kirromycin (kirromycin-Δ*kirOI*, **4**), and **5** were analysed at the Institute of Organic Chemistry at Tübingen University. NMR spectra were recorded on Bruker Avance III HDX 700 spectrometer (1 H, 700 MHz; 13 C, 150.8 MHz) with a 5 mm dual (1 H/13 C)-TCI cryo probe head at 298 K. Chemical shifts were given in ppm downfield from TMS relative to the solvent as internal standard (CD_3_OD, δ_H_ 3.30, δ_C_ 49.0 ppm).

## Results

### Sequence analysis of genes and encoded products presumably involved in kirromycin biosynthesis in *S*. *collinus* Tü 365

The genes *kirM*, *kirHVI*, *kirOI*, *kirOII*, *kirHIV*, *kirHV*, and *kirN* are located in the kirromycin gene cluster and thus a potential function of their gene products was postulated based on sequence analysis using the sequence aligner DIAMOND^19^ against the MIBiG database (Table 1).

**Table 1:** Results of the sequence analysis of KirM, KirHVI, KirOI, KirOII, KirHIV, KirHV, and KirN

Gene	nt/aa^1^	MIBiG (1)/UniProt KB/Swiss-Prot (2)	Highest similarity	Putative function	S/I^2^ (%)	Closest homologue in
*kirM*	957/319	1	*rapM*	SAM-dependent *O-*methyltransferase	87/75	*Streptomyces hygroscopicus* NRRL 5491
*kirHVI*	846/282	1	*fum3p*	Hydroxylase	55/31	*Gibberella fujikuroi*
*kirOI*	1200/400	1	*tiaP2*	Cytochrome P450 hydroxylase	64/49	*Dactylosporangium aurantiacum* subsp. *hamdenensis* NRRL 18085
*kirOII*	1218/406	1	*eryF*	Cytochrome P450 hydroxylase	59/40	*Saccharopolyspora erythraea* NRRL 2338
*kirHIV*	783/261	2	A0A062 WMT4	Uncharacterised protein	88/76	*Frankia* sp. BMG5.23
*kirHV*	396/132	2	A0A1Q5MN63	Uncharacterised protein	80/71	*Streptomyces* sp. CB00455
*kirN*	1368/456	1	*sfaR*	Crotonyl-CoA reductase/carboxylase	86/76	*Streptomyces flaveolus* DSM 9954

^1^nt, nucleotides; aa, amino acids. ^2^S, similarity; I, identity.

For the tailoring enzymes, the closest homologue of KirM was the *O-*methyltransferase RapM (75% aa identity and 87% similarity), involved in rapamycin biosynthesis in *Streptomyces hygroscopicus* NRRL 5491^20^. With 31% aa identity and 55% similarity, the hydroxylase Fum3p involved in C-5 hydroxylation in fumonisin biosynthesis in *Gibberella fujikuroi*^21^ was the best hit for KirHVI. When the protein sequence of KirHVI was aligned against the Uniprot/Swissprot database, most of the hits were members of the phytanoyl-CoA dioxygenases enzyme family. KirOI and KirOII were putatively assigned to cytochrome P450 hydroxylases. KirOI displayed 49% aa identity and 64% similarity to the previously characterised cytochrome P450 hydroxylase TiaP2, responsible for hydroxylation of the C-20 residue in tiacumicin B biosynthesis^22^. For KirOII, the closest characterised homologue (40% aa identity and 59% similarity) was found to be the 6-deoxyerythronolide B hydroxylase, encoded by *eryF*, which is involved in biosynthesis of erythromycin in *Saccharopolyspora erythraea*^23^.

Additional proteins downstream of the PKS/NRPS biosynthetic core have remained assigned with hypothetical function as it is the case for KirHIV and KirHV. The sequence analyses of KirHIV and KirHV, using DIAMOND^19^ alignment against the MIBiG database, did not yield any significant hits. Furthermore, based on the alignment against the UniProtKB/Swiss-Prot database, KirHIV and KirHV displayed similarity only to proteins with uncharacterised functions. Here, the best match for KirHIV (76% aa identity and 88% similarity) was an uncharacterised protein found in *Frankia* sp. BMG5.23. KirHV displayed the highest aa identity and similarity (71 and 80%) to an uncharacterised protein from *Streptomyces* sp. CB00455. Hence, in respect of the sequence analyses the functions of KirHIV and KirHV remained elusive.

The gene *kirN* is located downstream of the PKS I/NRPS-encoding region in the BGC and shows 76% aa identity and 86% similarity to the crotonyl-CoA reductase/carboxylase (CCR) SfaR from the sanglifehrin BGC in *Streptomyces flaveolus* DSM 9954^24^ (Table 1). In addition to this, the sequence analysis revealed that *kirN* displays between 70 to 76% aa identity with several other CCRs found in other *Streptomyces* species.

### Gene inactivations of *kirM*, *kirHVI*, *kirOI*, and *kirOII* result in production of kirromycin derivatives

To determine the role of the six genes presumed to be involved in tailoring reactions, two different gene replacement strategies were carried out. For the mutants in *kirM*, *kirHVI*, *kirHIV*, and *kirHV*, the respective gene was replaced by the ermE* promoter, whereas for *kirOI* and *kirOII*, a thiostrepton resistance cassette together with the ermE* promoter were used to replace the genes. Mutants were verified by control PCRs (see Supplementary Fig. S6), which resulted in either the expected bands or no product when the primers targeted internal regions of the deleted gene. The confirmed mutant clones were subjected to fermentation experiments carried out in parallel batches with wild type *S*. *collinus* Tü 365 as reference strain. The cultures were extracted with ethyl acetate, concentrated, and the extracts, including kirromycin and its derivatives, were dissolved in methanol. The samples were analysed by HPLC-HRMS.

**Figure 2:**
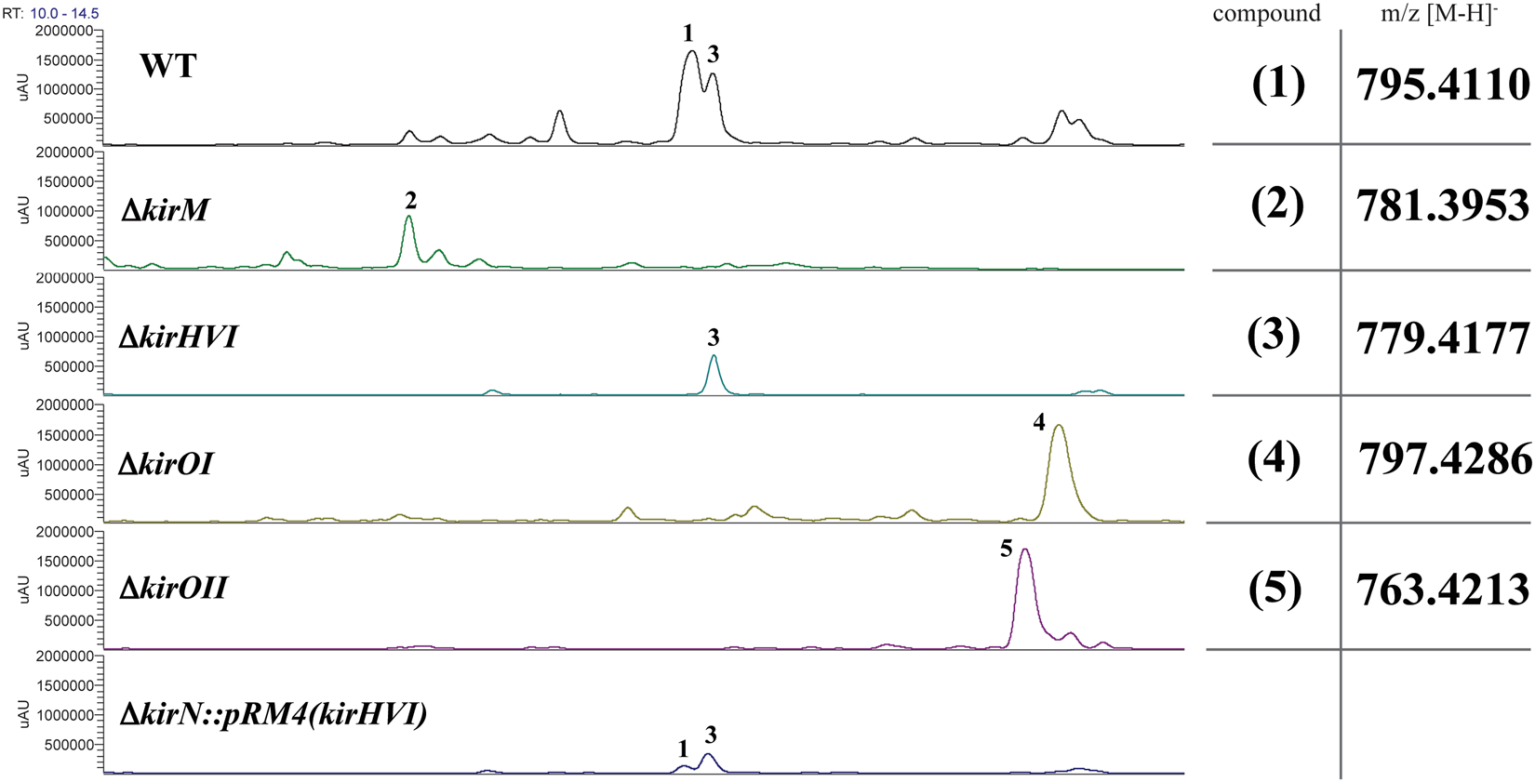
HPLC UV-Vis chromatograms and m/z values for kirromycin (1), produced by the wild type strain (WT), and derivatives produced by the mutants Δ*kirM* (2), Δ*kirHVI* (3), Δ*kirOI* (4), Δ*kirOII* (5), and Δ*kirN*::pRM4(*kirHVI*).

In the 30 min chromatographic method used for separation of kirromycin (**1**) and its derivatives, **1** appeared at 12.2 min with the main ion m/z = 795.4110 [M-H]^−^ (Fig. 2). Furthermore, MS/MS fragmentation of the derivatives was carried out to gain insights into differences in the chemical formulas (see Supplementary Fig. S2). Kirromycin derivatives were detected in the mutants of Δ*kirM*, Δ*kirHVI*, Δ*kirOI*, and Δ*kirOII* (see Fig. 2 and Supplementary Table S3). Gene inactivations of the hypothetical proteins encoded by *kirHIV* and *kirHV* did not affect the production of kirromycin and resulted in HPLC-HRMS data similar to the wild type (see Supplementary Fig. S1-I and S1-II). Thus, it was concluded that these proteins are not directly involved in kirromycin biosynthesis.

Gene inactivation of *kirM* resulted in the complete loss of production of **1**, and instead the derivative 20-*O*-demethyl-kirromycin (kirromycin-Δ*kirM*, **2**), which was only present in low amounts in the wild type sample, was the main product of the mutant. With its chemical formula C_42_H_58_N_2_O_12_, **2** appeared in the chromatogram at 11.2 min with m/z = 781.3953 [M-H]^−^ (Fig. 2 and Supplementary Table S3). The MS/MS fragmentation of **2** gave rise to a fragment ion of m/z = 485.2292 [M-H]^−^ (see Supplementary Fig. S2-II). Compared to the fragment ion of **1**, which was m/z = 499.2451 [M-H]^−^, the difference of - Δ14 corresponds to a difference of CH_2_. This is in accordance with the predicted mass of a methyl group and gives strong evidence that KirM catalyses the *O-*methylation at the C-20 position in the kirromycin precursor.

Similar to the *∆kirM* mutant, the extracts of *∆kirHVI*, *∆kirOI*, and *∆kirOII* were analysed using HPLC-HRMS and HRMS/MS. The chromatograms of these mutants revealed new prominent peaks, which were only detected with weak intensities in the extract of the wild type *S*. *collinus* Tü 365. Gene inactivation of *kirHVI* led to the production of 30-deoxy-kirromycin (kirromycin-Δ*kirHVI*, **3**). Based on the HRMS data (Fig. 2 and Supplementary Table S3), the chemical formula of **3** was calculated to be C_43_H_60_N_2_O_11_ and the corresponding ion m/z = 779.4177 [M-H]^−^ appeared at 12.3 min. This derivative was already observed in the chromatogram of the WT. In this case, the presence of **3** in the wild type extract could be a pathway intermediate in the biosynthesis of kirromycin. MS/MS fragmentation patterns for **1** and **3** yielded the same fragment ion with m/z = 499.2457 [M-H]^−^ (see Supplementary Fig. S2-I and S2-III). This mass corresponds to a fragmentation at the peptide bond at C-27^25^. As this pyridone-containing fragment is observed in both **1** and **3** this indicated that the hydroxyl group is missing on the sugar-containing fragment, presumably at position C-30, of **3** in the Δ*kirHVI* mutant. ^1^H and ^13^C NMR and Homonuclear Correlation Spectroscopy (COSY), Heteronuclear Single Quantum Correlation (HSQC), and Heteronuclear Multiple Bond Correlation (HMBC) corroborated the putative proposed structure of **3** as the analogue 30-deoxy-kirromycin (see Supplementary Table S4, Supplementary Fig. S4-I and Fig. S5-I).

Gene inactivations of the putative P450-dependent hydroxylases encoded by *kirOI* and *kirOII* resulted in detection of the two derivatives 5,6-dihydro-kirromycin (kirromycin-Δ*kirOI*, **4**) and 30-hydroxy-5,6-dehydro-1-*N*-demethyl-16-deoxy-kirrothricin (kirromycin-Δ*kirOII*, **5**) with m/z 797.4286 [M-H]^−^ and 763.4213 [M-H]^−^, which eluted at 13.6 min and 13.5 min, respectively (Fig. 2 and Supplementary Table S3). The chemical formulas were predicted to be C_43_H_62_N_2_O_12_ for compound **4** and C_43_H_60_N_2_O_10_ for **5**. For **4**, the MS/MS fragmentation data allowed for the assignment of KirOI to the double bond between C-5 and C-6 in the pyridone ring. The fragmentation of **4** yielded the fragment ion of m/z = 501.2634 [M-H]^−^, which is the respective fragment of m/z = 499.2451 [M-H]^−^ of **1** (see Supplementary Fig. S2-I and S2-IV). The mass unit difference of + Δ2 could be assigned to the gain of two hydrogen atoms for **4** compared to **1**. 1D and Two-Dimensional (2D)-NMR analyses of **4** confirmed the two CH_2_ groups at C-5 and C-6 (see Supplementary Table S4, Supplementary Fig. S4-II and Fig. S5-II).

In the case of KirOII, the difference of two oxygen atoms underlined the hypothesis of the enzyme being involved in oxidation at C-16 and presumably preparing the ring closure to the THF ring. However, the MS/MS fragmentation of **5**, yielding the main fragment ion of m/z = 467.2548 [M-H]^−^, did not allow for prediction of the exact chemical structure of the derivative (see Supplementary Fig. S2-V). Therefore, NMR analyses, including ^1^H NMR, ^13^C NMR, COSY, HSQC, and HMBC were undertaken to gain more insight into the structure of **5** (see Supplementary Table S4, Supplementary Fig. S4-III, and Fig. S5-III). To elucidate the structure of **5**, a 2 L-fermentation of the Δ*kirOII* mutant strain was carried out. Pure compound **5** (2.2 mg) was obtained by separation on a semi-preparative RP-18 column by HPLC. The ^13^C NMR spectrum showed addition signals for double bonds and the loss of signals for three oxygen-bearing carbons indicating opening of the furan ring and corroborated the chemical nomenclature 30-hydroxy-5,6-dehydro-1-*N*-demethyl-16-deoxy-kirrothricin. 2D-NMR experiments revealed the tetraene fragment for **5**, which is not present in **1** (see Supplementary Table S4 and Supplementary Fig. S4-III and S5-III).

To rule out polar effects caused by disruption of the kirromycin BGC, gene complementations for each of the mutants, Δ*kirM*, Δ*kirHVI*, and Δ*kirOII*, were carried out using conjugation and the integrative plasmid pRM4^18^, and for Δ*kirOI* using the replicative plasmid pGM1190 (see Supplementary Fig. S3-I–S3-IV). The complemented mutants were fermented in kirromycin production medium, extracted, and the extracts analysed by HPLC-HRMS. The complementation of the Δ*kirM* mutant resulted in similar kirromycin production levels compared to the wild type (100%). For the complementation of the Δ*kirHVI* mutant, approximately 30% of the wild type levels of **1** could be restored, which was confirmed by both MS and UV-Vis quantification. The complementation of the Δ*kirOI* and Δ*kirOII* mutants resulted in only 10% and 20% restored production of **1** compared to the wild type levels. Although there was no full complementation for these two mutants, the retention times and molecular masses, as determined by HPLC-HRMS, clearly indicated that the observed effects were due to the inactivation of the individual genes and not due to polar effects on the biosynthetic assembly line.

### Gene inactivation of *kirN* gives rise to lowered kirromycin production

The Δ*kirN* mutant was constructed based on the previously described GusA system for double crossover^14^. An ermE* promoter was used to replace the gene. The integrative plasmid pRM4 was used to construct the complemented mutant. The gene *kirN* encodes a putative CCR, which is postulated to provide the ethylmalonyl-CoA extender unit for the *trans-*AT KirCII, which then loads this substrate onto KirAII-ACP5. Unexpectedly, a deletion of *kirN* (Δ*kirN*) resulted in loss of kirromycin production. Instead, derivative **3**, which was the main compound observed in the Δ*kirHVI* mutant, was detected. Genetic complementation of the Δ*kirN* mutant failed to restore production of **1**. However, upon gene complementation of the Δ*kirN* mutant with pRM4 harbouring *kirHVI*, levels of **1** were partially restored (~30% of wild type levels). Since the replacement of *kirN* with the ermE* promoter was expected to drive expression through the downstream gene *kirHVI*, we examined the genetic organisation. This revealed overlapping open reading frames (ORFs) of *kirN* and *kirHVI* and it was concluded that the genetic inactivation of *kirN* led to the disruption of downstream gene *kirHVI*. To test if production of **1** could be fully restored, pRM4 harbouring a copy of both *kirN* and *kirHVI* was constructed and used for complementation of Δ*kirN*. This resulted in a restoration of **1** in the Δ*kirN* mutant, reaching between 40–100% of wild type levels (see Supplementary Fig. S3-V and S3-VI). Taken together, this data confirmed that the downstream gene *kirHVI* was affected by the gene inactivation of *kirN* and a full recovery of the production of **1** was only possible when *kirN* and its neighbouring gene *kirHVI* were encoded on the complementation construct.

## Discussion

In this study, the involvement of the seven genes, *kirM*, *kirHVI*, *kirOI*, *kirOII*, *kirHIV*, *kirHV*, and *kirN* in the biosynthesis of kirromycin in *S*. *collinus* Tü 365 was investigated experimentally based on gene inactivations, complementations, and product analyses by HPLC-HRMS, MS/MS, and NMR.

Our studies provide experimental evidence that KirM acts as an *O*-methyltransferase, giving rise to the methyl group on O-42 in kirromycin. This finding is further strengthened by comparison of the role of the close homologue RapM, which is involved in rapamycin biosynthesis in *Streptomyces hygroscopicus* NRRL 5491. Gene complementation with *rapM* of the deletion mutant MG2–10 (Δ*rapKIJMNOQL*) resulted in the restored *O-*methylation at the C-16 position in this molecule^20^.

Based on the MS/MS fragmentation (see Supplementary Fig. S2-III) and NMR analysis (see Supplementary Table S4, Supplementary Fig. S4-I and Fig. S5-I), the main derivative **3**, produced by the Δ*kirHVI* mutant, lacks a hydroxyl group at position C-30 in the kirromycin analogue. From the sequence-based alignment against the MIBiG database, KirHVI displays similarity to the hydroxylase Fum3p, which is responsible for attachment of a hydroxyl group at the C-5 position in fumonisin B produced by *Gibberella fujikuroi*^21^. Heterologous expression of Fum3p in *Saccharomyces cerevisiae* could clarify the enzymatic function of the protein and based on *in vitro* and *in vivo* findings it was possible to determine Fum3p to be a 2-ketoglutarate-dependent dioxygenase^26^. Since most protein hits from the UniProtKB/Swiss-Prot sequence alignment of KirHVI belonged to the family of phytanoyl-CoA dioxygenases it can be postulated that the hydroxylation catalysed by KirHVI in kirromycin biosynthesis occurs in a similar fashion (see Supplementary Fig. S7-I).

Furthermore, the function of KirOI and KirOII has been analysed in this study. According to the data from the MS/MS fragmentation and NMR, the Δ*kirOI* mutant produced the derivative kirromycin-Δ*kirOI*, which was undoubtedly missing the double bond between C-5 and C-6 in the pyridone ring (see Supplementary Table S4, Supplementary Fig. S2-IV, Fig. S4-II, and Fig. S5-II). Here, the predicted cytochrome P450 hydroxylase KirOI most likely forms a hydroxylated intermediate, which then undergoes dehydrogenation giving rise to the fully unsaturated pyridone ring (see Supplementary Fig. S7-II).

The HRMS and MS/MS analyses of the derivative **5**, produced by the Δ*kirOII* mutant, did not provide sufficient data for unambiguous structure prediction. Therefore, **5** was subjected to NMR studies, which enabled us to uncover a kirromycin analogue missing the THF ring structure and both hydroxyl groups at positions C-15 and C-16 (see Supplementary Table S4, Supplementary Fig. S4-III, and Fig. S4-III) in the kirromycin analogue. Based on our current hypothesis, the biosynthesis of kirromycin from **5** might be explained by a mechanism similar to that of the cytochrome P450 monooxygenase AurH, which is involved in the conversion of deoxyaureothin to aureothin through the formation of two C-O bonds, ultimately giving rise to a THF ring^27,28^. Similar to AurH, KirOII could introduce two hydroxyl groups at C-14 and C-16. Allylic substitution of the hydroxyl system could then lead to the formation of the THF ring system at the expense of H_2_O. The proposed enzymatic pathway is presented in Supplementary Fig. S7-III. In the future, enzymatic *in vitro* assays are necessary to fully establish the enzymatic reaction catalysed by KirOII.

Finally, we have studied the putative CCR encoded by *kirN*, which is expected to be involved in biosynthesis of the ethylmalonyl-CoA extender unit incorporated in kirromycin at the C-28 position^4^. Studies on the involvement of CCRs in both primary and secondary metabolism have led to a revision of their function and today these enzymes are known for their involvement in acetyl-CoA assimilation through an alternative pathway distinct from the glyoxylate pathway^29,30^. Furthermore, sequence analysis of actinomycetes has revealed a tendency of co-clustering of CCRs with PKSs. This suggests an important role of the CCRs in polyketide derivatization as they provide extender units alternative to butyrate, which are incorporated into the polyketide structure^30,31^. The putative role of KirN is further strengthened by the ^13^C NMR studies on the *N*-methylated kirromycin analogue aurodox, in which Liu *et al*.^32,33^ confirmed the incorporation of an intact butyrate unit at the C-28 position in the molecule. We suspect that the presence of an additional *ccr* gene in the genome of *S*. *collinus* Tü 365 (locus B446_29770 in NCBI GenBank entry CP006259.1), likely involved in the ethylmalonyl pathway to assimilate C2-units in primary metabolism^29^, may give rise to an inherent complementation of Δ*kirN*. Therefore, complete abolishment of kirromycin production was not expected. Surprisingly, the Δ*kirN* mutant generated in this study gave rise to production of **3** instead of the expected lowered production of **1**. Examination of the genetic organisation of the kirromycin BGC revealed the ORF of *kirN* to overlap with that of *kirHVI*, hence leading to disruption of the latter gene and production of **3**. Partial and full restoration of kirromycin production was achieved by genetic complementation of Δ*kirN* with *kirHVI* separately and with *kirN* and *kirHVI* together, respectively (see Supplementary Fig. S3-V and S3-VI). The observed phenomenon should be kept in mind for future BGC engineering efforts to account for polar effects in these often tightly regulated systems.

**Figure 3:**
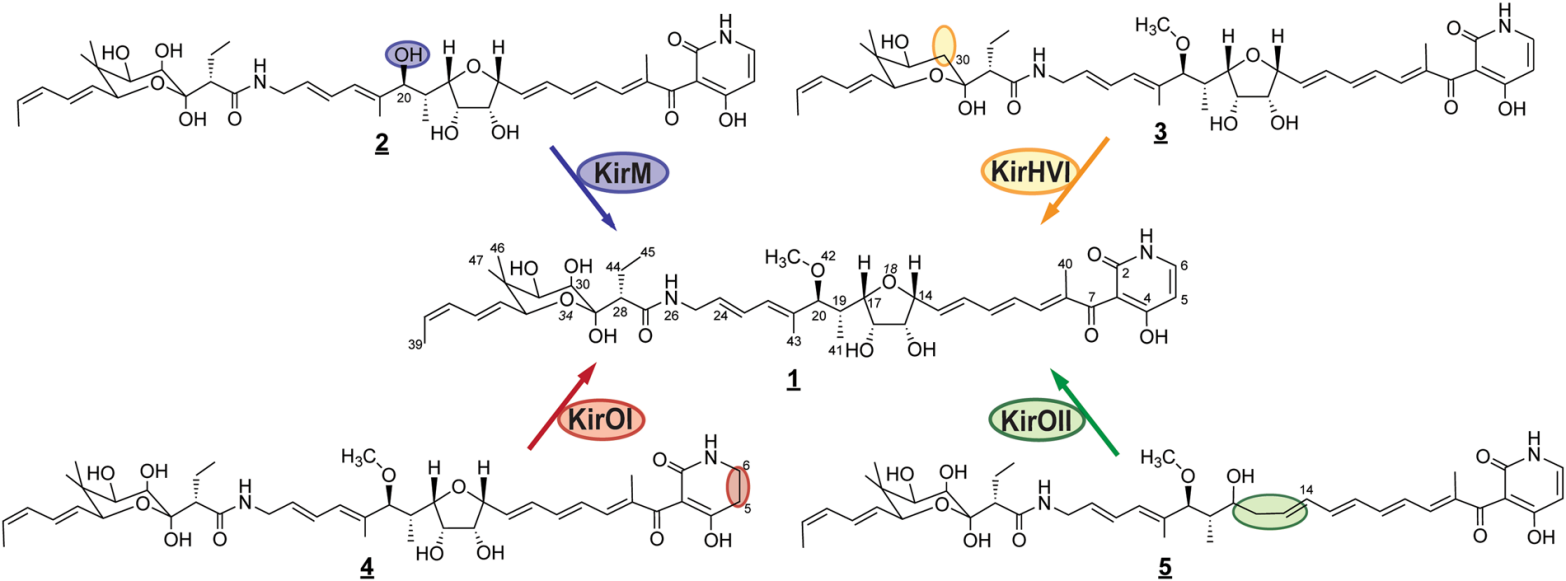
Structures of the new kirromycin derivatives produced by the mutants Δ*kirM*, Δ*kirHVI*, Δ*kirOI*, and Δ*kirOII*.

In our study, we demonstrated that the enzymes KirM, KirHVI, KirOI, and KirOII play important roles in late stages of the biosynthesis of kirromycin. Derivatives of **1** were detected in all mutants, except for Δ*kirN*, Δ*kirHIV*, and Δ*kirHV*. Although the genes *kirHIV* and *kirHV* are located in the kirromycin BGC, their inactivation had no effect on kirromycin biosynthesis and resulted in similar production profiles in both the wild type and mutant clones. Consequently, the data suggest that these hypothetical proteins are not directly involved in kirromycin biosynthesis. For the Δ*kirN* mutant, we observed a reduced production of **1**. While the Δ*kirM* mutant produces a derivative missing a methyl group at the O-42 position, the main derivative produced by the Δ*kirHVI* mutant lacks the hydroxyl group at the C-30 position in the sugar-like moiety. Finally, gene inactivations of *kirOI* and *kirOII*, encoding the two cytochrome P450 hydroxylases, result in a missing double bond in the pyridone ring and no THF ring closure, respectively. These results and the new knowledge have allowed us to close some of the gaps in the biosynthetic pathway of kirromycin (Fig. 3). Our studies does not allow for determining a defined sequence of the tailoring reactions. However, based on our findings, it could be argued that the enzymes act in an independent manner.

The better understanding of the complex kirromycin assembly line, which can be regarded as model for hybrid *cis*-/*trans*-AT PKS I/NRPSs, provides new incentive to the development of novel strategies for the production of polyketide derivatives. In particular the Δ*kirN* mutant, generated and analysed in this study, will support the applicability of a previously developed system^25^. In a previous study, we demonstrated that the promiscuity of the *trans*-AT KirCII, which also accepts non-natural malonate-derived extender units, can be used as a tool for polyketide diversification. By expressing a tailored malonyl-CoA synthetase and feeding of allyl- or propargylmalonic acid externally, the production of allyl- and propargyl-kirromycin was achieved^25^. However, the reported yields of propargyl-kirromycin were low compared to the production of the wild type kirromycin (22% propargyl-kirromycin compared to 78% kirromycin), likely due to the higher activity of KirCII towards the native substrate ethylmalonyl-CoA^34^. Hence, the use of the *ΔkirN* mutant with its impaired ethylmalonyl-CoA biosynthetic pathway is expected to result in improved yields of the novel derivatives.

## Electronic supplementary material

Supplementary Information
