# Supplementary material for: Filling the Gaps in the Kirromycin Biosynthesis: Deciphering the Role of Genes Involved in Ethylmalonyl-CoA Supply and Tailoring Reactions
Source: Sci Rep. 2018 Feb 19;8:3230. doi: 10.1038/s41598-018-21507-6 (PMC5818483; doi:10.1038/s41598-018-21507-6)
Supplement: Supplementary file 1 — Supplementary Information [file 41598_2018_21507_MOESM1_ESM.pdf]

Supplementary Information

Filling the Gaps in the Kirromycin Biosynthesis: Deciphering the Role of Genes Involved in Ethylmalonyl-CoA Supply and Tailoring Reactions

Helene L. Robertsen[a,1], Ewa M. Musiol-Kroll[a,1,2,3], Ling Ding[1], Kristina J. Laiple[2], Torben Hofeditz[4], Wolfgang Wohlleben[2,3], Sang Yup Lee[1,5], Stephanie Grond[4], and Tilmann Weber[1]

[a]Equal contribution

[1]Novo Nordisk Foundation Center for Biosustainability, Technical University of Denmark, Kemitorvet 220, 2800 Kgs. Lyngby, Denmark

[2]Eberhard-Karls-Universität Tübingen, Interfakultäres Institut für Mikrobiologie und Infektionsmedizin, Microbiology/Biotechnology, Auf der Morgenstelle 28, 72076 Tübingen, Germany

[3]German Centre for Infection Research (DZIF), Partner site Tübingen, Auf der Morgenstelle 28, 72076 Tübingen, Germany

[4]Eberhard-Karls-Universität Tübingen, Institut für Organische Chemie, Auf der Morgenstelle 18, 72076 Tübingen, Germany

[5]Department of Chemical and Biomolecular Engineering (BK21 Plus Program), Korea Advanced Institute of Science and Technology (KAIST), 291 Daehak-ro, Yuseong-gu, Daejeon 305-701, Republic of Korea

Table of Contents

Table S1: Strains and plasmids in this study..........3

Table S2: Primers used in this study..........6

Table S3: HPLC-ESI-HRMS data for kirromycin and derivatives..........9

Table S4: ^{1}H NMR spectra and assignments..........10

Figure S1-I – S1-II: UV-VIS profiles of Δ*kirHIV* and Δ*kirHV* mutants..........14

Figure S2-I – S2-V: MS/MS spectra of kirromycin and derivatives..........15

Figure S3-I – S3-VI: HPLC-ESI-UV-Vis profiles of the wild type (WT), Δ*kir* mutants, and complemented mutants..........18

Figure S4-IA/B – S4-IIA/B: ^{1}H NMR and ^{13}C NMR data and assignment for 30-deoxy-kirromycin (3), 5,6-dihydro-kirromycin (4), and 30-hydroxy-5,6-dehydro-1-*N*-demethyl-16-deoxy-kirrothricin (5)..........21

Figure S5-I – S5-III: 2D-NMR of 30-deoxy-kirromycin (3), 5,6-dihydro-kirromycin (4), and 30-hydroxy-5,6-dehydro-1-*N*-demethyl-16-deoxy-kirrothricin (5)..........25

Figure S6-I – S6-II: Verification of Δ*kir* mutants by control PCRs..........35

Figure S7-I – S7-III: Putative enzymatic reactions catalysed by tailoring enzymes KirHVI, KirOI, and KirOII..........36

Supplementary references..........38

Table S1: Strains and plasmids in this study

Source	Description/Genotype	Reference
***E. coli* strains**		
E. coli DH5-α	*fhu*A2, Δ(*arg*F-*lac*Z)U169, *pho*A, *gln*V44, Φ80, Δ(*lac*Z)M15, *gyr*A96, *rec*A1, *rel*A1, *end*A1, *thi*-1, *hsd*R17	New England Biolabs
E. coli ET12567 (pUZ8002)	*dam*13::Tn9(ChlR), *dcm*-6, *hsd*M, *hsd*R, *rec*F143, *zjj*-201::Tn10, *gal*K2, *gal*T22, *ara*14, *lac*Y1, *xyl*-5, *leu*B6, *thi*-1, *ton*A31, *rps*L136, *his*G4, *tsx*-78, *mtl*I, *gln*V44. pUZ8002(KanR)	1
***Streptomyces* strains**		
S. collinus Tü 365	Wild type *Streptomyces collinus* Tü 365	2
Δ*kirM*	*S. collinus* Tü 365 deletion mutant in *kirM*	This study
Δ*kirHVI*	*S. collinus* Tü 365 deletion mutant in *kirHVI*	This study
Δ*kirOI*	*S. collinus* gene replacement mutant in which *kirOI* is replaced by the thiostrepton resistance cassette with the *ermE** promoter downstream. ThioR	This study
Δ*kirOII*	*S. collinus* gene replacement mutant in which *kirOII* is replaced by the thiostrepton resistance cassette with the *ermE** promoter downstream. ThioR	This study
Δ*kirHIV*	*S. collinus* Tü 365 deletion mutant in *kirHIV*	This study
Δ*kirHV*	*S. collinus* Tü 365 deletion mutant in *kirHV*	This study
Δ*kirN*	*S. collinus* Tü 365 deletion mutant in *kirN*	This study
Δ*kirOI*-pGM1190	Δ*kirOI* complemented with replicative plasmid pGM1190-*kirOI*. ApraR, ThioR	This study
Δ*kirM*-pRM4	Δ*kirM* complemented with integrative plasmid pRM4-*kirM*. ApraR	This study
Δ*kirHVI*-pRM4	Δ*kirHVI* complemented with integrative plasmid pRM4-*kirHVI*. ApraR	This study
Δ*kirOII*-pRM4	Δ*kirOII* complemented with integrative plasmid pRM4-*kirOII*. ApraR, ThioR	This study
Δ*kirN*-pRM4_1	Δ*kirN* complemented with integrative plasmid pRM4-*kirHVI*. ApraR	This study
Δ*kirN*-pRM4_2	Δ*kirN* complemented with integrative plasmid pRM4-*kirN+HVI*. ApraR	This study
**Cosmids**		
1C24	pOJ436 cosmid vector with a fragment of the genome of kirromycin producer *Streptomyces collinus* Tü 365	3
2K05	pOJ436 cosmid vector with a fragment of the genome of kirromycin producer *Streptomyces collinus* Tü 365	3
**Plasmids**		
pJet1.2/blunt	*rep*(pMB1), *bla*(AmpR), *eco*47IR, PlacUV5, T7 RNA polymerase promoter. Part of the CloneJET™ PCR Cloning Kit used for high efficiency cloning of PCR products	Thermo Fisher Scientific Inc.
pDrive	T7 RNA polymerase promoter, LacZα, SP6 RNA polymerase promoter, AmpR, KanR, pUC origin, phage f1 origin of replication. For construction of Δ*kirOI* and Δ*kirOII* gene inactivation plasmids	Qiagen
pGUSA21	Promoter probe vector, pSETGUS with deleted KpnI fragment containing TipA promoter. *gusA*, Δ*int*, Δ*attB*, MCS from pUC21. Used for gene inactivation	4

Source	Description/Genotype	Reference
pGM1190	pGM190 derivative in which the kanamycin resistance cassette is exchanged with the apramycin resistance gene *aac3*(3)IV. TipA promoter. ApraR, ThioR. Used for complementation of the Δ*kirOI* mutant	G. Muth (personal communication)
pRM4	pSET152*ermEp* derivative with an artificial RBS, ΦC31 integration vector, *ermE** promoter. ApraR. Used for complementation of the *kir* mutants	[5]
pA18mob	pK18mob derivative in which *aph*II is replaced with the apramycin resistance cassette. *ori*T, *lacZ*α. ApraR. Used for construction of gene replacement mutants of *kirOI* and *kirOII*.	[3]
pSLE61	pUC19 derivative. *lacZ*α, *ori*I, pSG5 replicon. AmpR, ThioR. For amplification of thiostrepton resistance cassette	[6]
pCRISPR-Cas9	Derivative of pGM1190 with Cas9 under the control of TipA promoter	[7]
pCRISPR-Cas9_USER-*ΔkirM*	USER-compatible destination vector, which is a derivative of pGM1190 with Cas9 under the control of TipA promoter. Cloned with *ermE** promoter between sgRNA and 1 kb flanking regions right and left of *kirM* inserted in the USER cassette. ApraR	[7,8]
pCRISPR-Cas9_USER-*ΔkirHIV*	USER-compatible destination vector, which is a derivative of pGM1190 with Cas9 under the control of TipA promoter. Cloned with *ermE** promoter between sgRNA and 1 kb flanking regions right and left of *kirHIV* inserted in the USER cassette. ApraR	[7,8]
pCRISPR-Cas9_USER-*ΔkirHV*	USER-compatible destination vector, which is a derivative of pGM1190 with Cas9 under the control of TipA promoter. Cloned with *ermE** promoter between sgRNA and 1 kb flanking regions right and left of *kirHV* inserted in the USER cassette. ApraR	[7,8]
pCRISPR-Cas9_USER-*ΔkirN*	USER-compatible destination vector, which is a derivative of pGM1190 with Cas9 under the control of TipA promoter. Cloned with *ermE** promoter between sgRNA and 1 kb flanking regions right and left of *kirN* inserted in the USER cassette. ApraR	[7,8]
pHR1	pGUSA21 deletion plasmid of *kirM* cloned with *ermE** promoter between 1 kb flanking regions of the gene. 2.1 kb construct cloned from pCRISPR-Cas9_USER-*kirM*. ApraR	This study
pHR2	pGUSA21 deletion plasmid of *kirHVI* cloned with *ermE** promoter between 1 kb flanking regions of the gene. ApraR	This study
pHR3	pGUSA21 deletion plasmid of *kirHIV* cloned with *ermE** promoter between 1 kb flanking regions of the gene. 2.1 kb construct cloned from pCRISPR-Cas9_USER-*kirHIV*. ApraR	This study
pHR4	pGUSA21 deletion plasmid of *kirHV* cloned with *ermE** promoter between 1 kb flanking regions of the gene. 2.1 kb construct cloned from pCRISPR-Cas9_USER-*kirHV*. ApraR	This study
pHR5	pGUSA21 deletion plasmid of *kirN* cloned with *ermE** promoter between 1 kb flanking regions of the gene. 2.1 kb construct cloned from pCRISPR-Cas9_USER-*kirN*. ApraR	This study
pTL-*kirOI*	1.2 kb flanking regions left and right of *kirOI* cloned in pA18mob. ApraR	This study
pTL-*kirOI*-thio	pTL-*kirOI* cloned with thiostrepton resistance cassette	This study

Source	Description/Genotype	Reference
	inserted in XbaI site. ApraR, ThioR	
pDW-*kirOII*	2 kb flanking regions left and right of *kirOII* cloned in pA18mob. ApraR	This study
pDW-*kirOII*-thio	pDW-*kirOII* cloned with thiostrepton resistance cassette inserted in the XbaI site. ApraR, ThioR	This study
pJet1.2-*kirM*	pJet1.2/blunt with *kirM* cloned in the MCS. AmpR	This study
pJet1.2-*kirHVI*	pJet1.2/blunt with *kirHVI* cloned in the MCS. AmpR	This study
pJet1.2-*kirOI*	pJet1.2/blunt with *kirOI* cloned in the MCS. AmpR	This study
pJet1.2-*kirOII*	pJet1.2/blunt with *kirOII* cloned in the MCS. AmpR	This study
pJet1.2-*kirN*	pJet1.2/blunt with *kirN* cloned in the MCS. AmpR	This study
pJet1.2-*kirN+HVI*	pJet1.2/blunt with *kirN+HVI* cloned in the MCS. AmpR	This study
pGM1190-*kirOI*	pGM1190 with *kirOI* cloned in HindIII + EcoRI site	This study
pRM4-*kirM*	pRM4 with *kirM* cloned in NdeI + HindIII site	This study
pRM4-*kirHVI*	pRM4 with *kirHVI* cloned in NdeI + HindIII site	This study
pRM4-*kirOII*	pRM4 with *kirOII* cloned in NheI + HindIII site	This study
pRM4-*kirN*	pRM4 with of *kirN* cloned in NdeI + HindIII site	This study
pRM4-*kirN+HVI*	pRM4 with *kirN+kirHVI* cloned in NdeI + HindIII site	This study

Table S2: Primers used in this study

ID	Primer name	Sequence (5' - 3')	PCR program
***kirM, kirN, kirHIV*, and *kirHV* inactivation mutants**			
KP1	kirM_pGUS_left_FW	CTGCAGACGCGTCGACGTCAAGC GACGAGGAGCTGGCG	1. 98°C, 0:30 min<br>2. 98°C, 0:10 min<br>3. 73°C, 0:30 min<br>4. 72°C, 1:00 min<br>5. 72°C, 5:00 min<br>(Steps 2-4 × 34)
KP2	kirM_pGUS_left_RV	ATTACCCTGTTATCCCTAGTTAGT ACGCGGGCCCCAGG	
KP3	kirN_pGUS_left_FW	GGAGCTCGAATTCGAAGCTTCTG CAGACGCGTCGACGTCATATGCG ACCCCGACCAGGA	1. 98°C, 0:30 min<br>2. 98°C, 0:10 min<br>3. 75°C, 0:30 min<br>4. 72°C, 1:00 min<br>5. 72°C, 5:00 min<br>(Steps 2-4 × 34)
KP4	kirN_pGUS_right_RV	GCGGGAAGCAGTGATAAGCATT ACCCTGTTATCCCTAGTTCTAGA ATCTCCGACGACGCGT	
KP5	kirHIV_pGUS_left_FW	GCAGACGCGTCGACGTCACTGCT CAAGTACTTCGACTCGAC	1. 98°C, 0:30 min<br>2. 98°C, 0:10 min<br>3. 70°C, 0:30 min<br>4. 72°C, 1:00 min<br>5. 72°C, 5:00 min<br>(Steps 2-4 × 34)
KP6	kirHIV_pGUS_right_RV	ATTACCCTGTTATCCCTAGTTTGA CCAGCGCCAGTTGTG	
KP7	kirHV_pGUS_left_FW	CTGCAGACGCGTCGACGTCAGCT CAGCTTCGCCGC	1. 98°C, 0:30 min<br>2. 98°C, 0:10 min<br>3. 73°C, 0:30 min<br>4. 72°C, 1:00 min<br>5. 72°C, 5:00 min<br>(Steps 2-4 × 34)
KP8	kirHV_pGUS_right_RV	ATTACCCTGTTATCCCTAGTTGA CGATGTCGGGATCCTCCC	
***kirHVI* inactivation mutant**			
KP9	kirHVI_pGUS_left_FW	GACGCGTCGACGTCACCGGAGTC GTGCTG	1. 98°C, 0:30 min<br>2. 98°C, 0:10 min<br>3. 72°C, 0:30 min<br>4. 72°C, 0:30 min<br>5. 72°C, 5:00 min<br>(Steps 2-4 × 34)
KP10	kirHVI_pGUS_left_RV	GTCAAGATCGACCGCTTGTTTCC CTTCCCTG	
KP11	kirHVI_pGUS_ermE_FW	CTACAGGGAAGGGAAACAAGCG GTCGATCTTGACG	1. 98°C, 0:30 min<br>2. 98°C, 0:10 min<br>3. 74°C, 0:10 min<br>4. 72°C, 1:00 min<br>5. 72°C, 5:00 min<br>(Steps 2-4 × 34)
KP12	kirHVI_pGUS_ermE_RV	CGACGGGCGGCTCGCCGTCGATC CTACCAA	
KP13	kirHVI_pGUS_right_FW	GGTAGGATCGACGGCGAGCCGC CCGTCG	1. 98°C, 0:30 min<br>2. 98°C, 0:10 min<br>3. 75°C, 0:30 min<br>4. 72°C, 0:30 min<br>5. 72°C, 5:00 min<br>(Steps 2-4 × 34)
KP14	kirHVI_pGUS_right_RV	ATTACCCTGTTATCCCTAGTTGG ACGCGACCAGGCCGC	

ID	Primer name	Sequence (5' - 3')	PCR program
***kirOI* and *kirOII* inactivation mutants**			
KP15	1ldOxy5	AACTGCAGTCAACATCACCTACGACC	1. 94°C, 2:00 min<br>2. 94°C, 1:15 min<br>3. 60°C, 1:30 min<br>4. 72°C, 1:30 min<br>5. 72°C, 5:00 min<br>(Steps 2-4 × 30)
KP16	1ldOxy3	AATCTAGACTCGTCGTAGCCGGTG	
KP17	1rdOxy5	AATCTAGAGTCGGCCAGCAACTGG	
KP18	1rdOxy3	AAGAATTCGAACACGTCGATGTGCG	
KP19	thio-up	ACTCTAGATCACTGACGAATCGAGGTCGAGGA	
KP20	thio-low	AATCTAGAGGCGAATACTTCATATGCGGGGAT	
KP21	leftOIIeco5	GAATTCGTGCCAGGAGGTGATCC	1. 94°C, 2:00 min<br>2. 94°C, 1:15 min<br>3. 60°C, 1:30 min<br>4. 72°C, 2:00 min<br>5. 72°C, 5:00 min<br>(Steps 2-4 × 30)
KP22	leftOIIxba3	TCTAGACTCGACGTCCGTCCAATC	
KP23	rightOIIxba5	TCTAGAGGCCCGCGCTTCCTGAAAG	
KP24	rightOIIhind3	AAGCTTCAGCGCGAACTGCGTCG	
**Complementation plasmids**			
KP27	kirN_NdeI_FW	AAACATATGCAAGACATCATCGACGCCG	1. 98°C, 0:30 min<br>2. 98°C, 0:10 min<br>3. 60°C, 0:30 min<br>4. 72°C, *<br>5. 72°C, 5:00 min<br>(Steps 2-4 × 34)
KP28	kirN_HindIII_RV	AAAAAGCTTTCATTTGTTTCCCTTCCCTGTAGG	
KP29	kirM_NdeI_FW	AAACATATGAGCCAACCCGATGTGATGACC	
KP30	kirM_HindIII_RV	AAAAAGCTTTCAGACTCGGACGGCGAC	
KP31	kirHVI_NdeI_FW	AAACATATGACCGACGAAGACCTCGTCACG	
KP32	kirHVI_HindIII_RV	AAAAAGCTTTCAACCGGCGCGCTCGG	
KP33	kirOI_ HindIII_pGM1190_FW	AAAAAGCTTGTGTCCGAGACCGTTCGTCCCG	
KP34	kirOI_ EcoRI_pGM1190_RV	AAAGAATTCTCACCATGTCACCGGCAGCTCG	
KP35	kirOII_NheI_FW	AAAGCTAGCGTGACCGGAACATTCGATTGGACG	
KP36	kirOII_HindIII_RV	AAAAAGCTTTCACACGAGCTGGACGGGCAG	
**Control PCRs and sequencing**			
KP37	kirLEFT_ pGUS_seq_FW	GCTCGAATTCGAAGCTTCTGCAG	1. 95°C, 3:00 min<br>2. 95°C, 0:30 min<br>3. 61°C, 0:30 min<br>4. 72°C, 2:15 min<br>5. 72°C, 5:00 min<br>(Steps 2-4 × 40)
KP38	kirRIGHT_ pGUS_seq_RV	GATAAGCATTACCCTGTTATCCCTAG	
KP39	apra-up	AGCTTCTCAACCTTGG	1. 94°C, 2:00 min<br>2. 94°C, 1:00 min<br>3. 50°C, 1:00 min<br>4. 72°C, 1:00 min<br>5. 72°C, 5:00 min<br>(Steps 2-4 × 30)
KP40	apra-low	TCCGCCAAGGCAAAGC	

ID	Primer name	Sequence (5' - 3')	PCR program
KP41	oxy1	GCAAAGGAGAGCGTTGTGTC	1. 94°C, 2:00 min<br>2. 94°C, 1:00 min<br>3. 60°C, 1:00 min<br>4. 72°C, 1:10 min<br>5. 72°C, 5:00 min<br>(Steps 2-4 × 30)
KP42	oxy2	TGGTCTCCGTTCACCATGTC	
KP43	kirOII_check_FW	GCGATTGGGGATCTTGGTGA	
KP44	kirOII_check_RV	ATGGAAACGGTGGTCACACG	
KP45	kirM_int-500bp_FW	CCACCCTGTCCGGTCGCGG	1. 95°C, 3:00 min<br>2. 95°C, 0:30 min<br>3. 64°C, 0:30 min<br>4. 72°C, 2:30 min<br>5. 72°C, 5:00 min<br>(Steps 2-4 × 40)
KP46	kirM_int-500bp_RV	GCATCGTCCGCAGCCGCTG	
KP47	kirN_int-500bp_FW	GTGTTCGCGTGGAACCCCGG	
KP48	kirN_int-500bp_RV	GGGATCCTCCCCACCCGTG	
KP49	kirHIV_int-500bp_FW	CCTGGTGGAGGCCGACAACG	
KP50	kirHIV_int-500bp_RV	CGCCTCGGCGAGTTCGGCG	
KP51	kirHV_int-500bp_FW	GCATGAACTCCGTGCCCCAGG	
KP52	kirHV_int-500bp_RV	GCCGCCCGGTCAGTGCACG	
KP53	kirHVI_int-500bp_FW	TGGAGGAGTTCCTGGACCCCG	
KP54	kirHVI_int-500bp_RV	CGCCGTGATACAGACCGCCG	
KP55	kirOI_int-500bp_FW	CGAGGAAGGCGGACCCACCG	
KP56	kirOI_int-500bp_RV	GCGTCTCCCTCGGTGAAGTGGTG	
KP57	kirOII_int-500bp_FW	GACGGACCTCGTCGCCGGG	
KP58	kirOII_int-500bp_RV	GAGCTGGATCTGGCCGTCCTTG	
KP59	pJet_check_FW	CGACTCACTATAGGGAGAGCGGC	1. 95°C, 3:00 min<br>2. 95°C, 0:30 min<br>3. 60°C, 0:30 min<br>4. 72°C, **<br>5. 72°C, 5:00 min<br>(Steps 2-4 × 40)
KP60	pJet_check_RV	AAGAACATCGATTTTCCATGGCAG	
KP61	pGM1190_seq_FW	CACGCGGAACGTCCGGG	
KP62	pGM1190_seq_RV	CCGCTGAAACTGTTGAAAGTTGTTTAGC	
KP63	pRM4_seq_FW	CAGTCACGACGTTGTAAAACGACGG	
KP64	pRM4_seq_RV	GGCACCGCGATGCTGTTGTG	

* 0:15 min for *kirHV*. 0:30 min for *kirM/kirHIV/kirHVI*. 0:45 min for *kirN/kirOI/kirOII*.

** 1:00 min for *kirM/kirHVI*. 1:30 min for *kirN/kirOI/kirOII*.

Table S3: HPLC-ESI-HRMS data for kirromycin and derivatives

Compound	RT (min)	m/z for [M-H$^-$]	Δm (ppm)	Chemical Formula	MS/MS fragment (m/z [M-H$^-$])	MS/MS fragment (m/z [M+H$^+$])
Kirromycin (**1**)*	12.2	795.4110	-2.6	$C_{43}H_{60}N_2O_{12}$	499.2451	501.2578
20-*O*-demethyl-kirromycin (**2, Δ*kirM***)	11.2	781.3953	0	$C_{42}H_{58}N_2O_{12}$	485.2292	487.2292
30-deoxy-kirromycin (**3, Δ*kirHVI***)	12.3	779.4177	2.3	$C_{43}H_{60}N_2O_{11}$	499.2457	501.2586
5,6-dihydro-kirromycin (**4, Δ*kirOI***)	13.6	797.4286	1.6	$C_{43}H_{62}N_2O_{12}$	501.2634	503.2768
30-hydroxy-5,6-dehydro-1-*N*-demethyl-16-deoxy-kirrothricin (**5, Δ*kirOII***)	13.5	763.4213	-2.7	$C_{43}H_{60}N_2O_{10}$	467.2548	469.2698

*Δ*kirN*(*kirHVI*-complemented), Δ*kirHIV,* and Δ*kirHV* had similar chromatograms and MS profiles to those of the wild type kirromycin (**1**), albeit production was lowered in Δ*kirN*(*kirHVI*-complemented).

Table S4: ^{1}H NMR spectra and assignments

^{1}H nuclear magnetic resonance (NMR) (**A**) and ^{13}C NMR data (**B**) for 30-deoxy-kirromycin (**3**, kirromycin-Δ*kirHVI*), 5,6-dihydro-kirromycin (**4**, kirromycin-Δ*kirOI*), and 30-hydroxy-5,6-dehydro-1-*N*-demethyl-16-deoxy-kirrothricin (**5**, kirromycin-*ΔkirOII*) in CD_3OD at resonance frequency [1)]600 resp. 150.8 MHz or [2)]700 resp. 176.1 MHz. The chemical shifts, obtained from NMR spectroscopy analysis, are represented by Δ ppm

	**30-deoxy-kirromycin**<br>(**3,** from Δ*kirHVI*)		**5,6-dihydro-kirromycin**<br>(**4,** from Δ*kirOI*)		**30-hydroxy-5,6-dehydro-1-N-demethyl-16-deoxy-kirrothricin** (**5**, from Δ*kirOII*)	
**Position**	**δ_H [ppm]** (Integral, Type, J in Hz)[2)]	**δ_C [ppm]**	**δ_H [ppm]** (Integral, Type, J in Hz)[1)]	**δ_C [ppm]**	**δ_H [ppm]** (Integral, Type, J in Hz)[2)]	**δ_C [ppm]**
**1**		-	-	-	-	-
**2**		164.5	-	n.d.	-	164.5
**3**	-	112.5	-	79.5[00]	-	112.72<br>112.64
**4**	-	169.4	-	169.8	-	169.18, 169.01
**5**	6.11, 1H, s, br	102.1	2.54, 1H, dd, *J* = 7.0 (br)	37.3 (br)[#]	6.12, 1H, d, *J* = 7.4	102.0
**6**	7.39, 1H, br	137.8	3.42, 1H, m, *J* = 7.0 (br)	37.9	7.40, 1H, d, *J* = 7.4	137.8
**7**	-	198.9	-	n.d.	-	198.55, 198.63
**8**	-	141.4	-	138.8[00]	-	142.9
**9**	6.94, 1H, br	142.5	6.20, m	134.3[00]	6.97, 1H, ddd, *J* = 11.4, 5.3, 1.2	143.3
**10**	6.69, 1H, b, dd, *J* = 12.5, 12.5	129.4	6.56, m[00]	129.3	6.73, 1H, dd, *J* = 11.4, 14.7	129.5
**11**	6.07, 1H, dd, *J* = 15.1, 7.2	137.0	6.56, m[00]	137.2[00]	6.67, 1H, d, *J* = 14.7, 10.7	142.3
**12**	6.45, 1H, dd, *J* = 15.0, 11.0	133.6	6.40, 1H, *J* = 11.5, 15.0	133.9 (br)	6.61, 1H, dd, *J* = 14.7, 10.7	142.5
**13**	6.52, 1H, m (with 24)	136.1	5.99, m	135.1 (br)	6.46, 1H, dd, *J* = 10.5, 5.9<br>6.44, 1H, dd, *J* = 10.6, 5.7	138.7<br>134.0
**14**	4.31, 1H, m	81.6	4.25, 1H, dd, *J* = 12.5	81.8	6.37, 0.6 H, dd, *J* = 14.7, 10.5	132.1
**15**	4.32, 1H, m	75.0	4.28, 1H, dd, *J* = 12.5	75.0	5.90, 0.4 H, dt, *J* = 14.9, 7.4;<br>6.26, 0.6H dd, *J* = 14.8, 10.4<br>(also 6.21, dd, *J* = 11.3, 11.3, 5.67, m,; 6.78, m)	136.02<br>133.50<br>(131.1, 132.2, 133.5)
**16**	4.19, 1H, dd, *J* = 4.4	74.0	4.17, 1H, dd, *J* = 4.4	74.0	2.25, m<br>2.40, m<br>2.43, m	40.0, 40.7, 34.8

	**30-deoxy-kirromycin** (<u>**3**</u>, from Δ*kirHVI*)		**5,6-dihydro-kirromycin** (<u>**4**</u>, from Δ*kirOI*)		**30-hydroxy-5,6-dehydro-1-N-demethyl-16-deoxy-kirrothricin** (<u>**5**</u>, from Δ*kirOII*)	
**Position**	**δH [ppm]** (Integral, Type, J in Hz)2)	**δC [ppm]**	**δH [ppm]** (Integral, Type, J in Hz)1)	**δC [ppm]**	**δH [ppm]** (Integral, Type, J in Hz)2)	**δC [ppm]**
**17**	3.71, 1H, dd, 7.3, 4.1	85.0	3.68, 1H, dd, *J* = 4.0, 7.5	84.9	4.06, 1H, m	70.9
**18**	-	-	-	-	-	-
**19**	2.20, 1H, m	36.8	2.16, 1H, ddq, *J* = 2.0, 7.0	36.8	1.69, 1H, m	40.8
**20**	3.34, 1H, d, 9.2	91.9	3.32, s	91.9	3.46, 1H, d, *J* = 10.1	90.0
**21**	-	136.1	-	136.0	-	136.7
**22**	5.69, 1H, dd, *J* = 15.0, 6.0	130.8	5.96, 1H, d (br), *J* = 10	130.9	5.99, 1H, d, *J* = 10.5	130.4
**23**	6.56, 1H, dd, *J* = 15.2, 11.0	128.0	6.49, 1H, m, *J* = 11.0, 16.0	128.3	6.51, 1H, dd, *J* = 15.2, 11.0	128.3
**24**	6.53, 1H, dd, *J* = 15.2, 11.0	128.2	5.67, 1H, m, *J* = 6.0, 16.0	131.0	5.66, 1H, m (with 35)	130.6
**25a**	3.90, 1H, dd, *J* = 15.8, 5.8	42.0	3.86, 1H, dd, *J* = 6.5, 15.0	42.0	3.89, 1H, dd, *J* = 15.8, 5.5	42.1
**25b**	3.95, 1H, dd, *J* = 15.8, 5.9		3.93, 1H, dd, *J* = 6.5, 15.0		3.95, 1H, dd, *J* = 15.8, 6.7	
**26-N**	-	-	-	-	-	-
**27**	-	176.9	-	177.9	-	177.9
**28**	2.39, 1H, dd, *J* = 11.1, 4.2	58.3	2.82, 1H, dd, *J* = 4.0, 11.0	52.5	2.84, 1H, dd, *J* = 11.0, 4.5	52.5
**29**	-	99.5	-	101.0	-	101.0
**30**	1.95, 1H, dd, *J* = 12.6, 4.6<br>1.48, 1H, dd, *J* = 12.6, 12.5	38.3	3.65, 1H, d, *J* = 4.0	71.4	3.68, 1H, d, *J* = 3.6	71.4
**31**	3.74, 1H, d, *J* = 12.5, 4.6	72.6	3.57, 1H, d, *J* = 4.0	74.0	3.60, 1H, d, *J* = 3.6	74.0
**32**	-	40.3	-	40.0	-	39.9
**33**	4.17, 1H, d, *J* = 6.0	77.7	4.21, 1H, d, *J* = 6.0	77.3	4.24, 1H, t, *J* = 5.8	77.3
**34-O**	-	-	-	-		
**35**	5.64, 1H, m (with 24)	130.3	5.62, 1H, dd, *J* = 6.0, 15.0	130.5	5.66, 1H, m (with 24)	130.6
**36**	6.55, 1H, m (with 24)	127.9	6.54, m	127.7	6.58, 1H, dd, *J* = 14.8, 11.8	127.7
**37**	6.01, 1H, ddd, *J* = 10.8, 10.8,	130.4	5.99, m	130.6	6.01, 1H, ddd, *J* = 10.8, 10.8, 1.5	130.5

	**30-deoxy-kirromycin** (**3**, from Δ*kirHVI*)		**5,6-dihydro-kirromycin** (**4**, from Δ*kirOI*)		**30-hydroxy-5,6-dehydro-1-N-demethyl-16-deoxy-kirrothricin** (**5**, from Δ*kirOII*)	
**Position**	**δ_H [ppm]** (Integral, Type, J in Hz)[2)]	**δ_C [ppm]**	**δ_H [ppm]** (Integral, Type, J in Hz)[1)]	**δ_C [ppm]**	**δ_H [ppm]** (Integral, Type, J in Hz)[2)]	**δ_C [ppm]**
	1.0					
**38**	5.49, 1H, dq, *J* = 11.0, 6.9	126.5	5.44, 1H, dq, *J* = 7.0, 11.0	126.4	5.49, 1H, dq, *J* = 11.0, 7.0	126.4
**39**	1.77, 3H, dd, *J* = 7.1, 1.5	13.7	1.73, 3H, dd, *J* = 2.0, 7.0	13.7	1.77, 3H, dm, *J* = 7.0	13.7
**40**	2.01, 3H,	11.7	1.95, s (br)	14.4 (br)	2.00, 2.04, 3H, d, *J* = 0.9	11.61, 11.68
**41**	0.84, 3H, d, *J* = 6.8	13.9	0.81, 1H, d, *J* = 7.0	13.9	0.69, 3H, d, *J* = 7.0	9.49, 9.57
**42**	3.18, 3H, s	56.2	3.15, 3H, s (sh)	56.2	3.17, 3H, s	56.32
**43**	1.69, 3H, s	11.2	1.66, 3H, s (sh)	11.2	1.63, 3H, s	11.0 (sh)
**44**	1.73, 2H, m	21.6	1.70, m	21.1	1.73, 2H, m	21.1
**45**	0.95, 3H, t, *J* = 7.3	12.4	0.92, 3H, t, *J* = 7.5	12.3	0.95, 3H, t, *J* = 7.4	12.3
**46**	0.77, 3H, s	12.4	0.89, 3H, s[00]	15.9	0.92, 3H, s	15.9
**47**	0.94, 3H, s	23.0	0.89, 3H, s[00]	24.6	0.92, 3H, s	24.6

*: ^{13}C shift determined by HSQC or HMBC. [#]: assigned due to HSQC correlations. [00]: not unambiguously assigned due to tautomerization, missing 2D correlations, or overlaying signals. n.d.: not detected. n.a.: not assigned.

Figure S1-I – S1-II: UV-VIS profiles of Δ*kirHIV* and Δ*kirHV* mutants

The Δ*kirHIV* and Δ*kirHV* mutants were grown in parallel with *Streptomyces collinus* Tü 365 (wild type) in kirromycin production medium and extracted with ethyl acetate. The extracts were dried, dissolved in methanol, and analysed using HPLC-ESI-HRMS. The recorded UV profiles of Δ*kirHIV* **(I)** and Δ*kirHV* **(II)** (black chromatograms) were similar to those of the wild type (red chromatograms). These results indicate that the two genes *kirHIV* and *kirHV* are not directly involved in the biosynthesis of kirromycin. In the case of Δ*kirHV* **(II)**, we suspect that the lower intensity of the peak at 12.3 min (compared to the wild type) could be explained from almost complete conversion of the pathway intermediate into kirromycin.

(I)

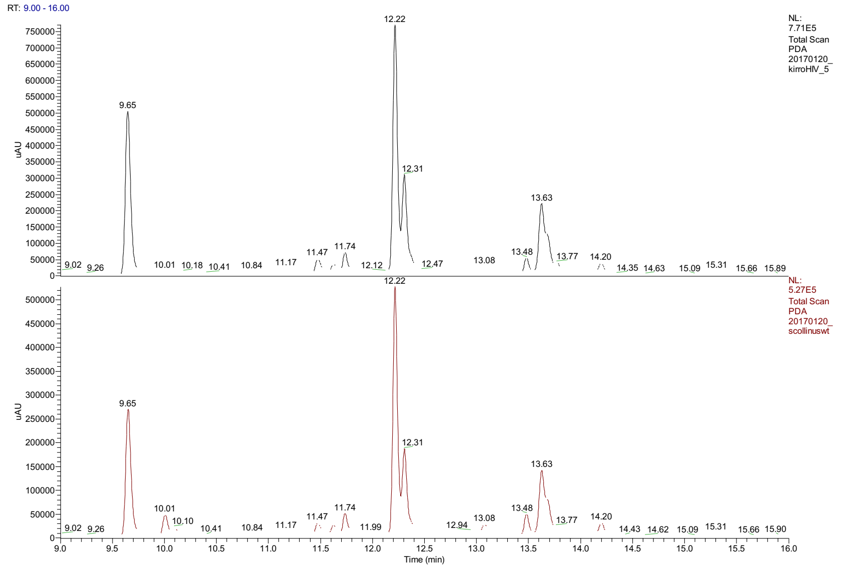

(II)

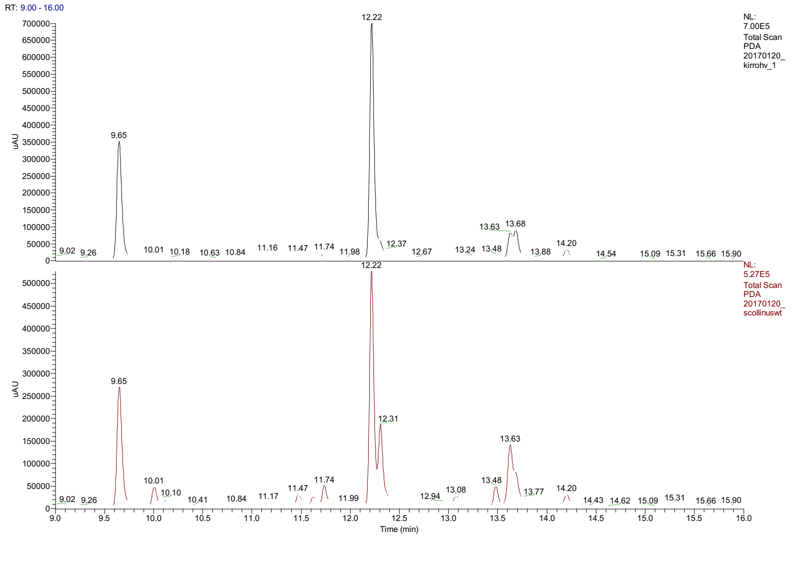

Figure S2-I – S2-V: MS/MS spectra of kirromycin and derivatives

MS/MS spectra for **(I)** kirromycin (**1**), **(II)** 20-*O*-demethyl-kirromycin (**2**), **(III)** 30-deoxy-kirromycin (**3**), **(IV)** 5,6-dihydro-kirromycin (**4**), and **(V)** 30-hydroxy-5,6-dehydro-1-*N*-demethyl-16-deoxy-kirrothricin (**5**). Recorded in negative mode (ESI-) with higher-energy collisional dissociation (HCD) of 25 eV.

(I)

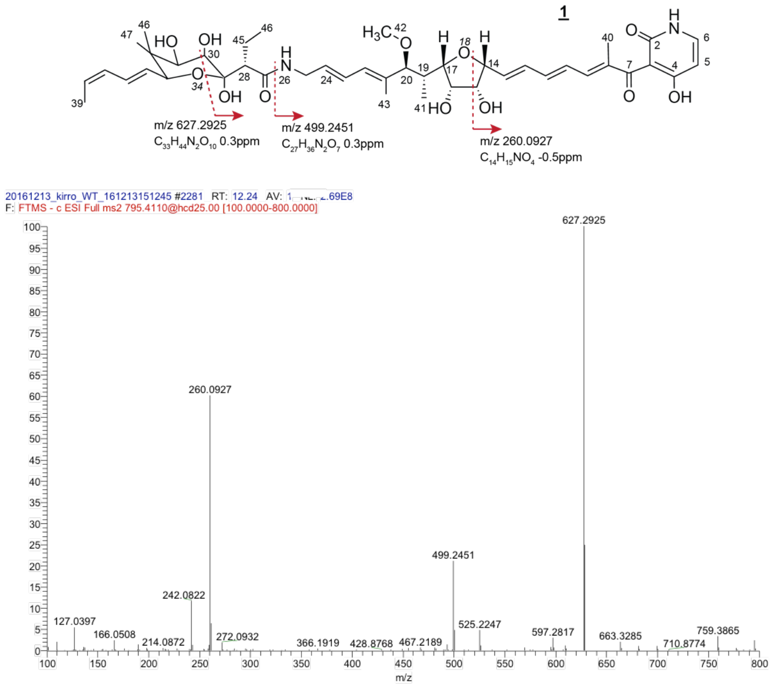

(II)

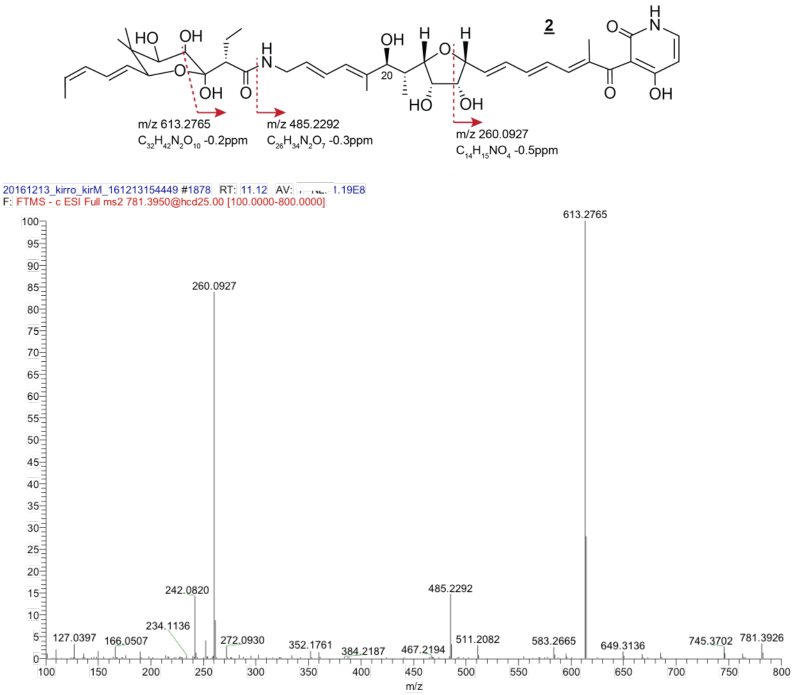

2
m/z 613.2765
C32H42N2O10 -0.2ppm
m/z 485.2292
C26H34N2O7 -0.3ppm
m/z 260.0927
C14H15NO4 -0.5ppm
20161213_kirro_kirM_161213154449 #1878 RT: 11.12
F: FTMS - c ESI Full ms2 781.3950@hcd25.00 [100.0000-800.0000]
613.2765
260.0927
242.0820
485.2292
234.1136
127.0397
166.0507
272.0930
352.1761
384.2187
467.2194
511.2082
583.2665
649.3136
745.3702
781.3926
m/z

(III)

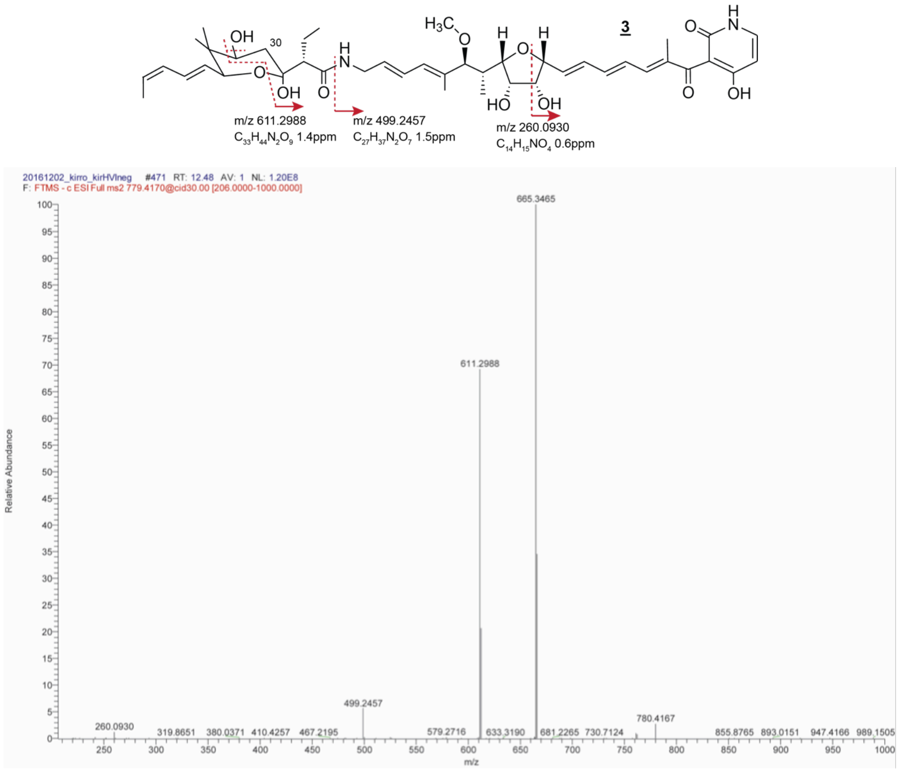

3
m/z 611.2988
C33H44N2O9 1.4ppm
m/z 499.2457
C27H37N2O7 1.5ppm
m/z 260.0930
C14H15NO4 0.6ppm
20161202_kirro_kirHVIneg #471 RT: 12.48 AV: 1 NL: 1.20E8
F: FTMS - c ESI Full ms2 779.4170@cid30.00 [206.0000-1000.0000]
665.3465
611.2988
499.2457
780.4167
Relative Abundance
m/z

(IV)

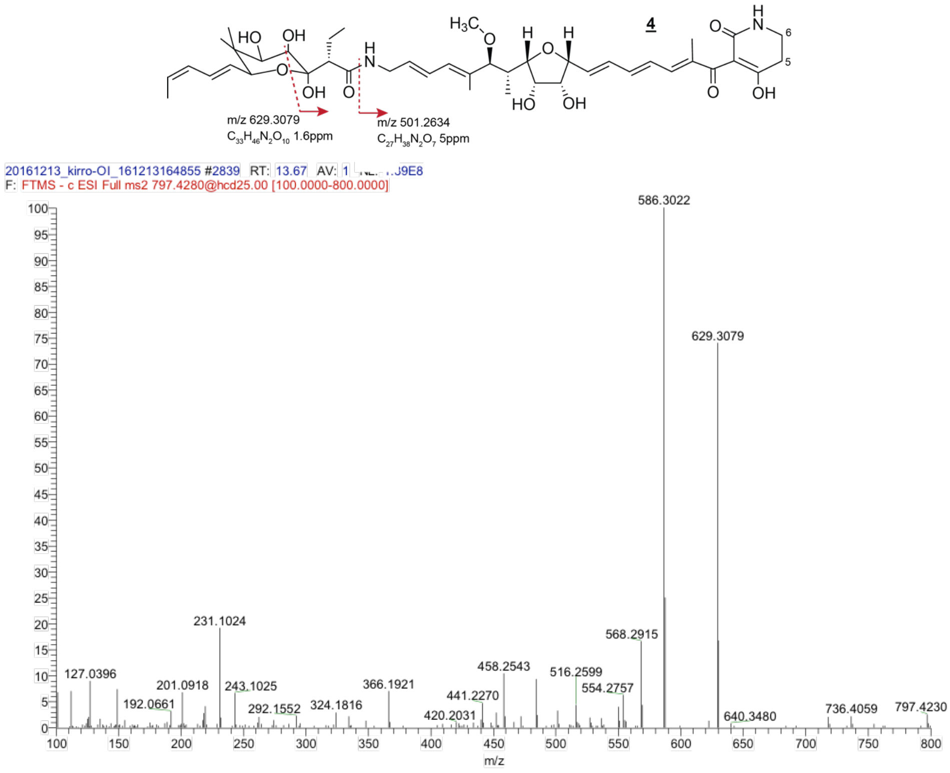
4
m/z 629.3079
$C_{33}H_{46}N_2O_{10}$ 1.6ppm
m/z 501.2634
$C_{27}H_{38}N_2O_7$ 5ppm
20161213_kirro-OI_161213164855 #2839 RT: 13.67
F: FTMS - c ESI Full ms2 797.4280@hcd25.00 [100.0000-800.0000]
586.3022
629.3079
568.2915
231.1024
127.0396
201.0918
243.1025
192.0661
292.1552
324.1816
366.1921
458.2543
441.2270
420.2031
516.2599
554.2757
640.3480
736.4059
797.4230
m/z

(V)

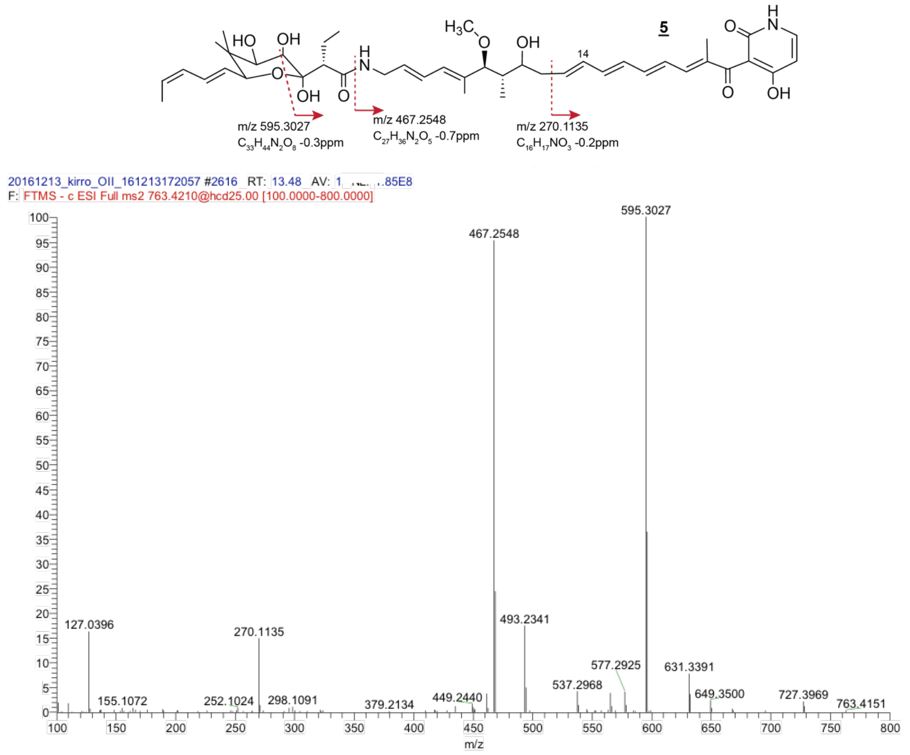
5
m/z 595.3027
$C_{33}H_{44}N_2O_8$ -0.3ppm
m/z 467.2548
$C_{27}H_{36}N_2O_5$ -0.7ppm
m/z 270.1135
$C_{16}H_{17}NO_3$ -0.2ppm
20161213_kirro_OII_161213172057 #2616 RT: 13.48
F: FTMS - c ESI Full ms2 763.4210@hcd25.00 [100.0000-800.0000]
595.3027
467.2548
493.2341
127.0396
270.1135
577.2925
631.3391
537.2968
155.1072
252.1024
298.1091
379.2134
449.2440
649.3500
727.3969
763.4151
m/z

Figure S3-I – S3-VI: HPLC-ESI-UV-Vis profiles of the wild type (WT), Δ*kir* mutants, and complemented mutants

UV-Vis profiles of *Streptomyces collinus* Tü 365 (WT) compared to mutants **(I)** Δ*kirM*, **(II)** Δ*kirHVI*, **(III)** Δ*kirOI,* and, **(IV)** Δ*kirOII*, along with their respective complementations. The Δ*kirN* mutant and its single (*kirHVI*) and double (*kirN+HVI*) complementations are shown in **(V)** and **(VI)**, respectively.

(I)

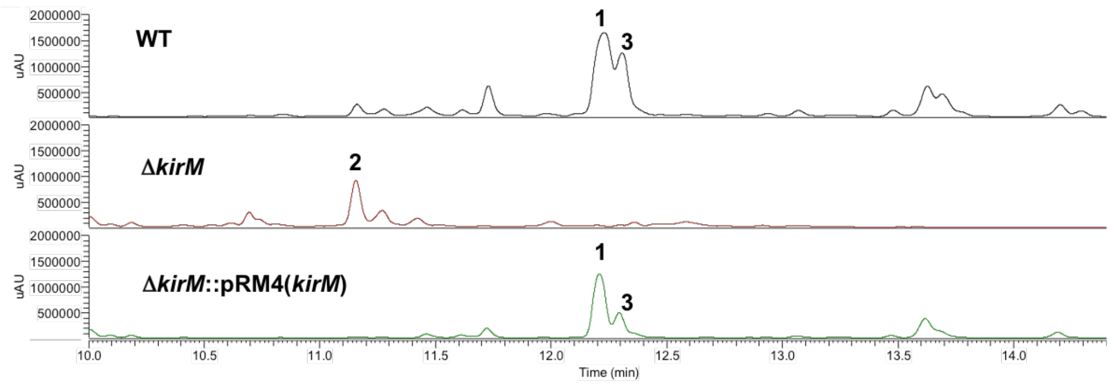

(II)

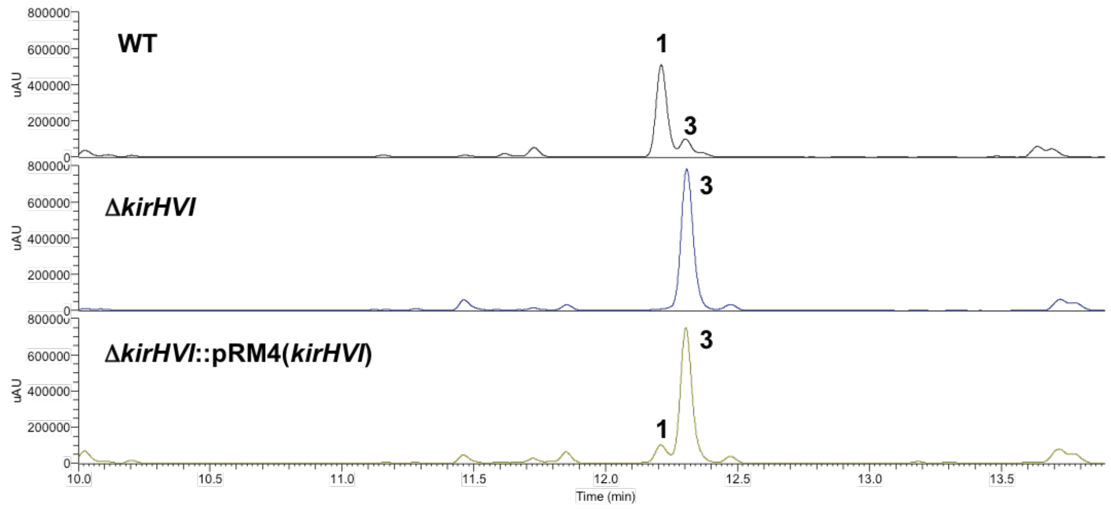

(III)

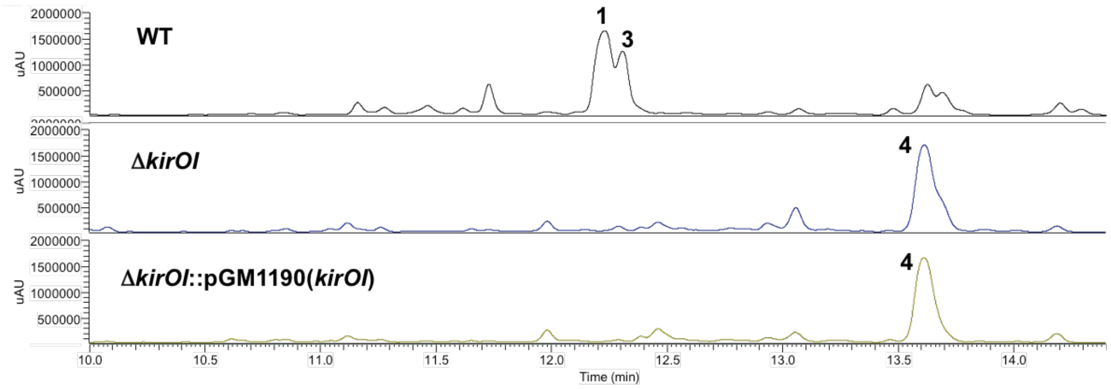

WT
1
3
ΔkirOI
4
ΔkirOI::pGM1190(kirOI)
4
uAU
2000000
1500000
1000000
500000
10.0
10.5
11.0
11.5
12.0
12.5
13.0
13.5
14.0
Time (min)

(IV)

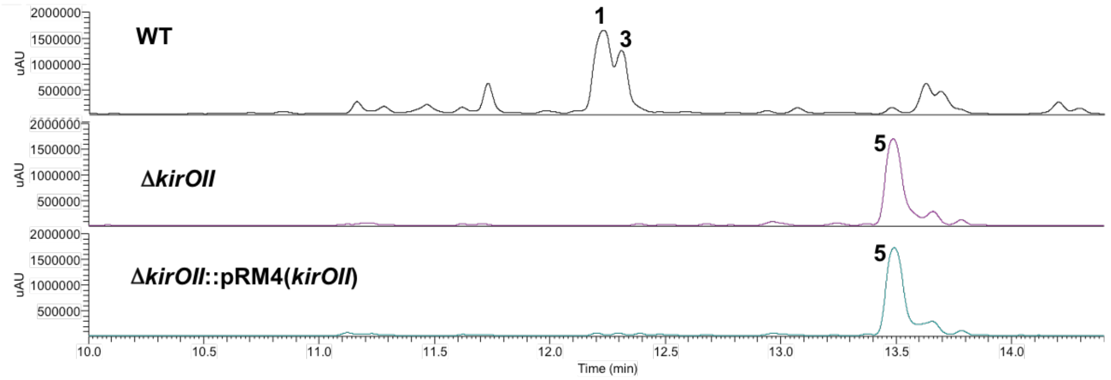

WT
1
3
ΔkirOII
5
ΔkirOII::pRM4(kirOII)
5
uAU
2000000
1500000
1000000
500000
10.0
10.5
11.0
11.5
12.0
12.5
13.0
13.5
14.0
Time (min)

(V)

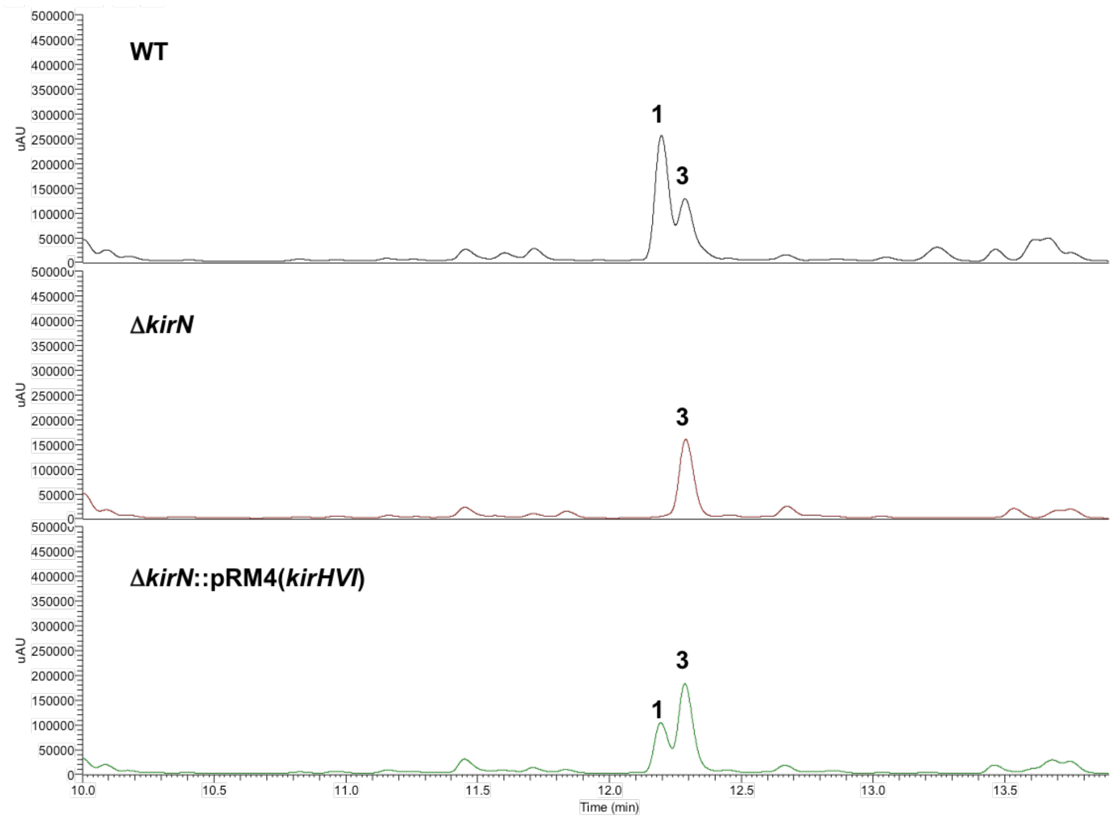

WT
1
3
ΔkirN
3
ΔkirN::pRM4(kirHVI)
1
3
uAU
Time (min)

(VI)

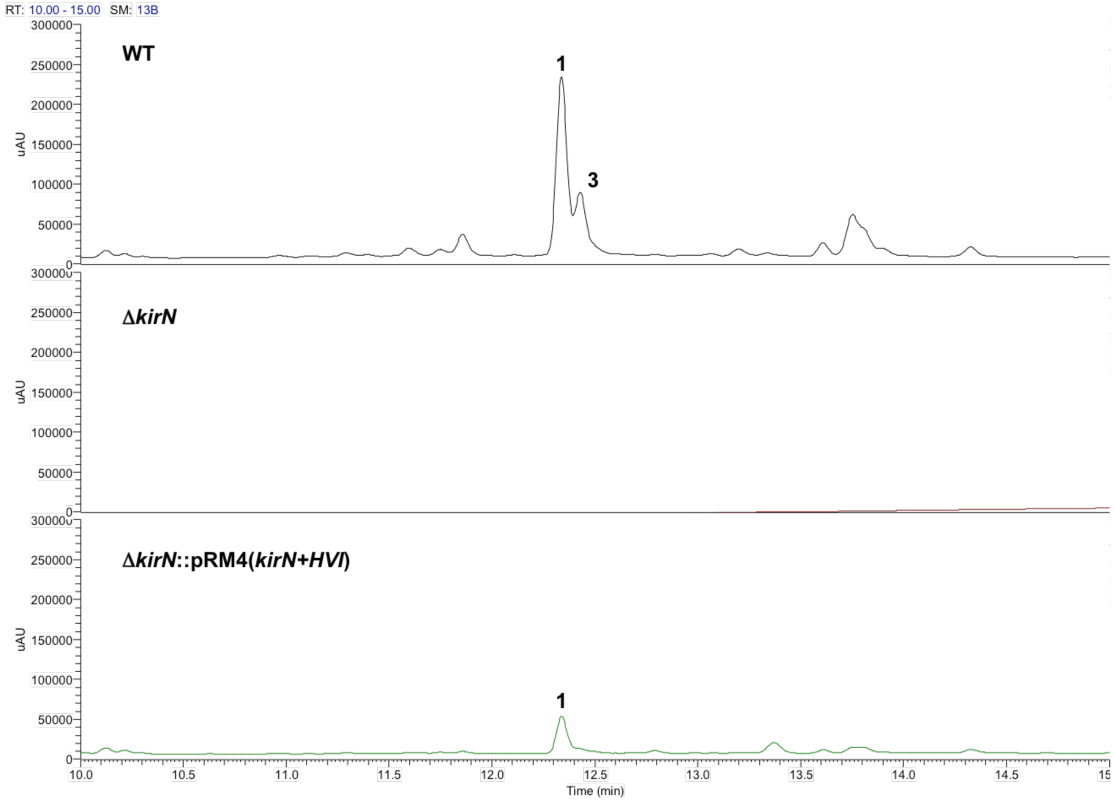

RT: 10.00 - 15.00 SM: 13B
WT
1
3
ΔkirN
ΔkirN::pRM4(kirN+HVI)
1
uAU
Time (min)

Figure S4-I – S4-III: ^{1}H NMR and ^{13}C NMR data and assignment for 30-deoxy-kirromycin (3), 5,6-dihydro-kirromycin (4), and 30-hydroxy-5,6-dehydro-1-*N*-demethyl-16-deoxy-kirrothricin (5)

(**A**) ^{1}H NMR and (**B**) ^{13}C NMR of (**I**) 30-deoxy-kirromycin (**3**), **(II)** 5,6-dihydro-kirromycin (**4**), and (**III**) 30-hydroxy-5,6-dehydro-1-*N*-demethyl-16-deoxy-kirrothricin (**5**) (CD_3OD, 298 K). Resonance frequency [1)] 600 resp. 150.8 MHz, [2)] 700 resp. 176.1 MHz.

(IA) ^{1}H NMR spectrum of 3

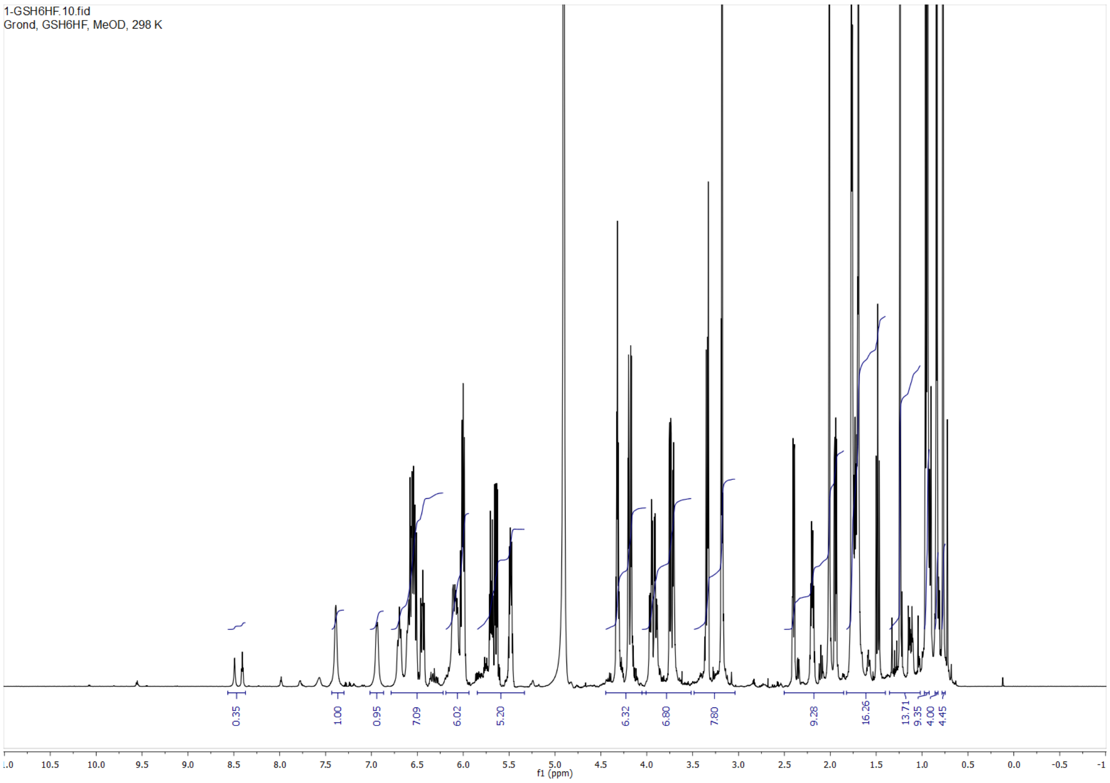

(IB) ^{13}C NMR spectrum of 3

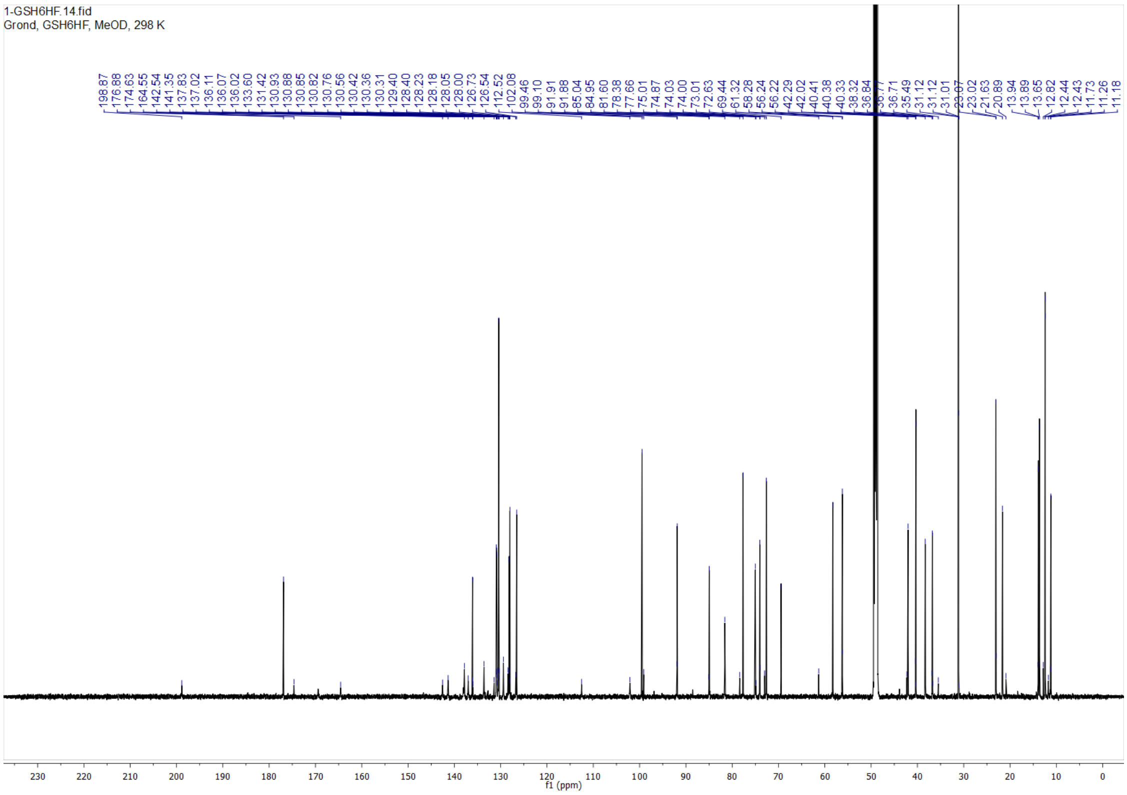

(IIA) ^{1}H NMR spectrum of 4

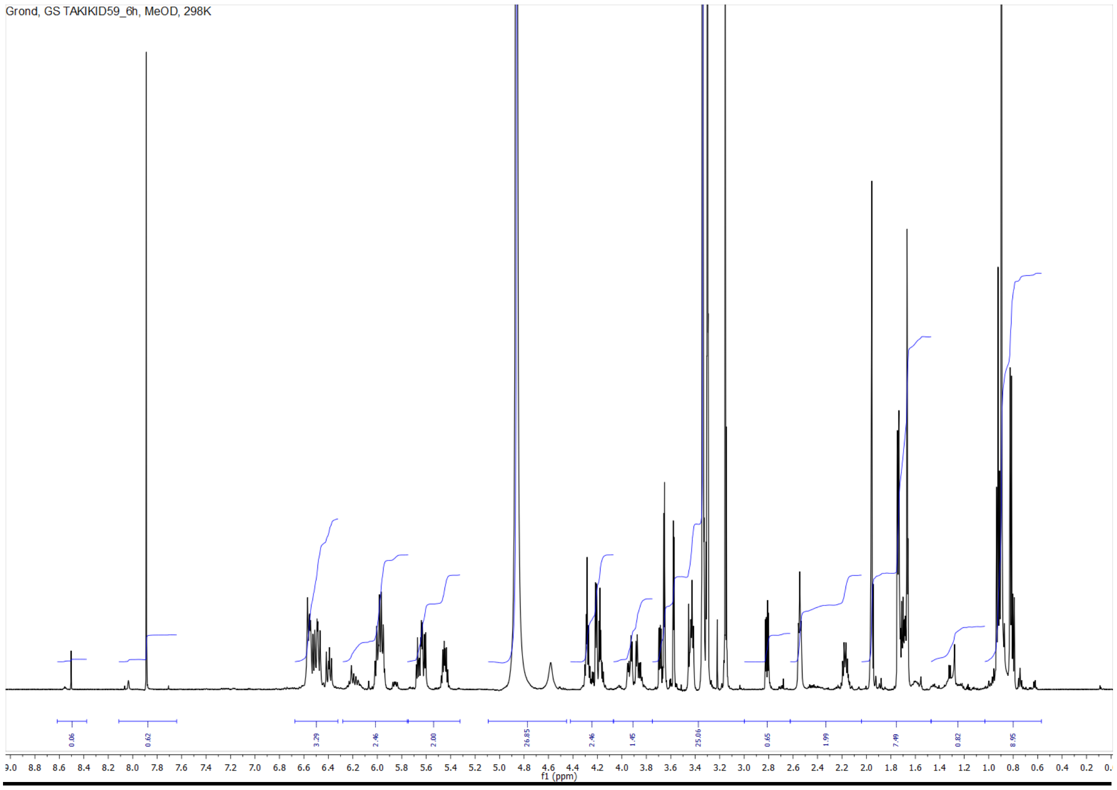

(IIB) ^{13}C NMR spectrum of 4

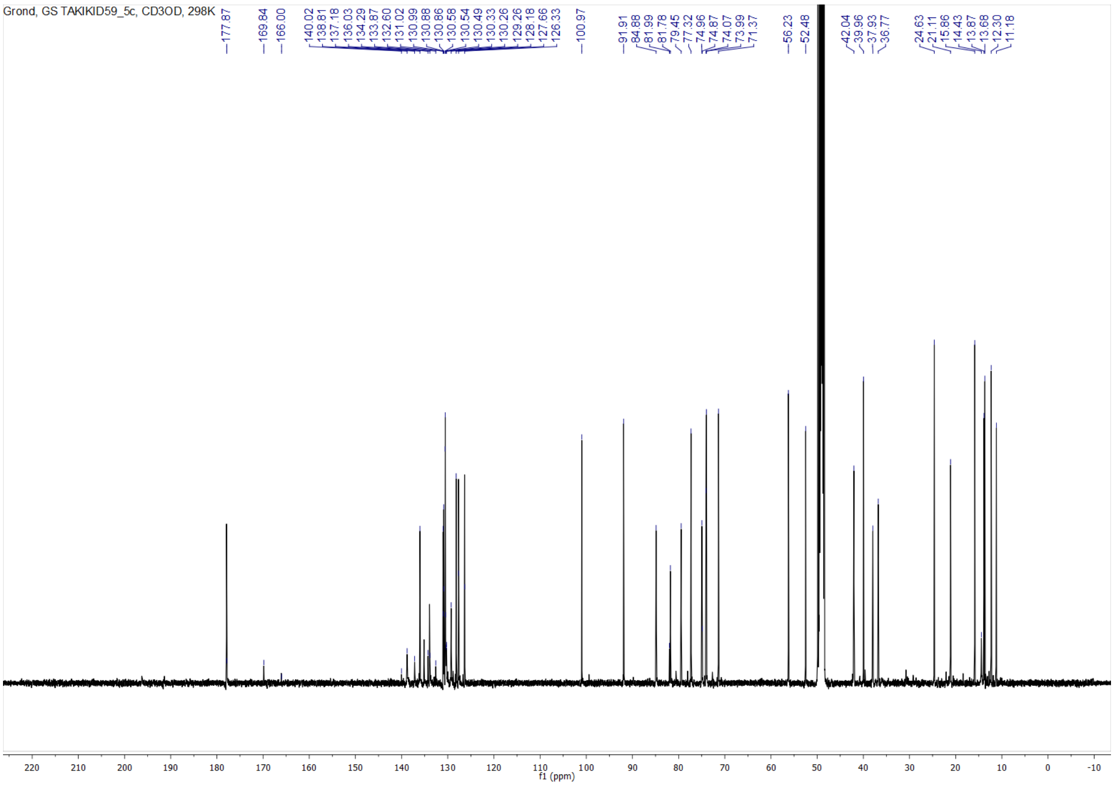

(IIIA) ^{1}H NMR spectrum of 5

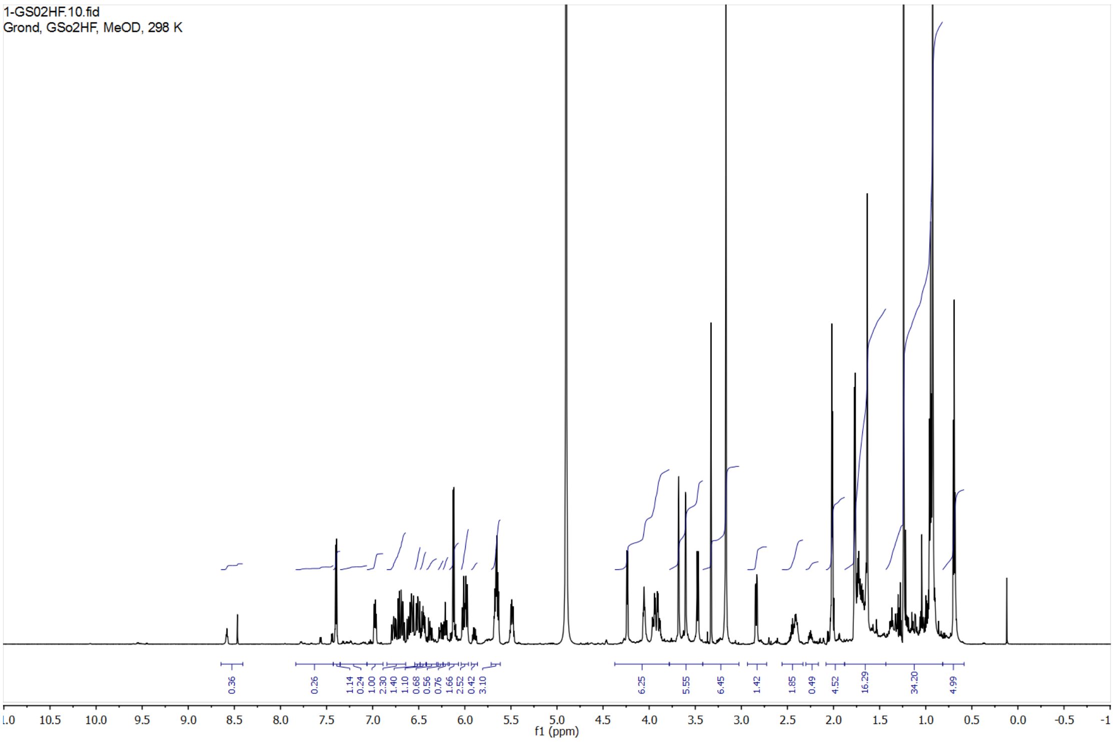

(IIIB) ^{13}C NMR spectrum of 5

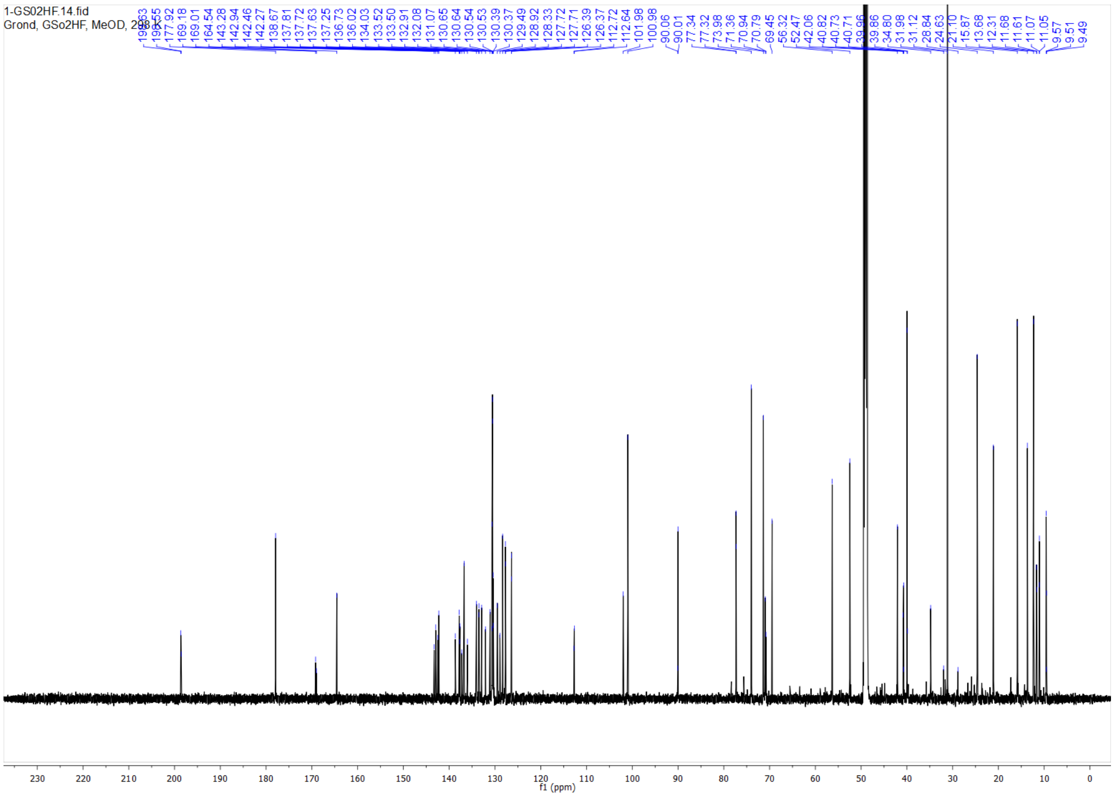

Figure S5-I – S5-III: 2D-NMR of 30-deoxy-kirromycin (3), 5,6-dihydro-kirromycin (4), and 30-hydroxy-5,6-dehydro-1-*N*-demethyl-16-deoxy-kirrothricin (5)

(I) 30-deoxy-kirromycin (3)

Homonuclear Correlation Spectroscopy (COSY) underlined the partial structure for the 30-deoxygenated pyran ring in 30-deoxy-kirromycin (**3**) (**IA** and **ID**), which was isolated from the Δ*kirHVI* mutant. It lacked the C-30-hydroxy group as had been found previously for the heneicomycin analogue[9]. The signal for H-30_a (δ 1.48) showed strong cross correlation with the H-30_b (δ 1.95), and both exhibited further correlations with H-31 (δ 3.74). **(IB)** The Heteronuclear Single Quantum Correlation (HSQC) gave evidence to the CH_2-connectivity for both H-30_a and H-30_b connection attached to one carbon atom (C-30, ($d_C = 38.3$). **(IC)** The Heteronuclear Multiple Bond Correlation (HMBC) spectrum showed correlations between H-30_a (δ 1.48), H-30_b (δ 1.95) and C-29 (δ 99.5) and corroborated the 30-deoxy ring in **3**.

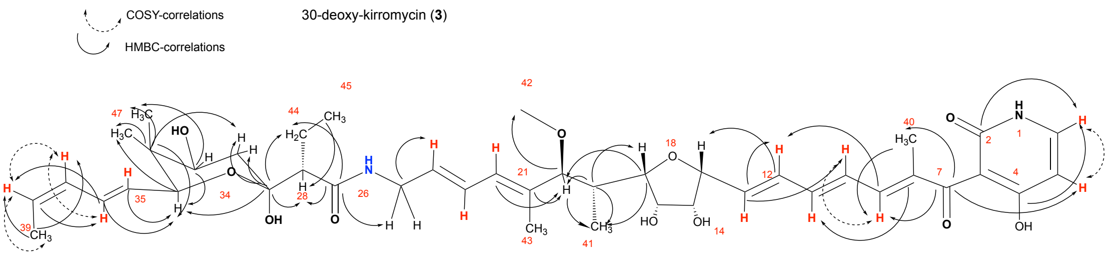

Formula S5-I Selected COSY (double dashed arrowed), TOCSY (dashed line), and HMBC (single arrowed) correlations for 30-deoxy-kirromycin (**3**).

(IA) H-H-correlation (COSY) spectrum of 30-deoxy-kirromycin (3)

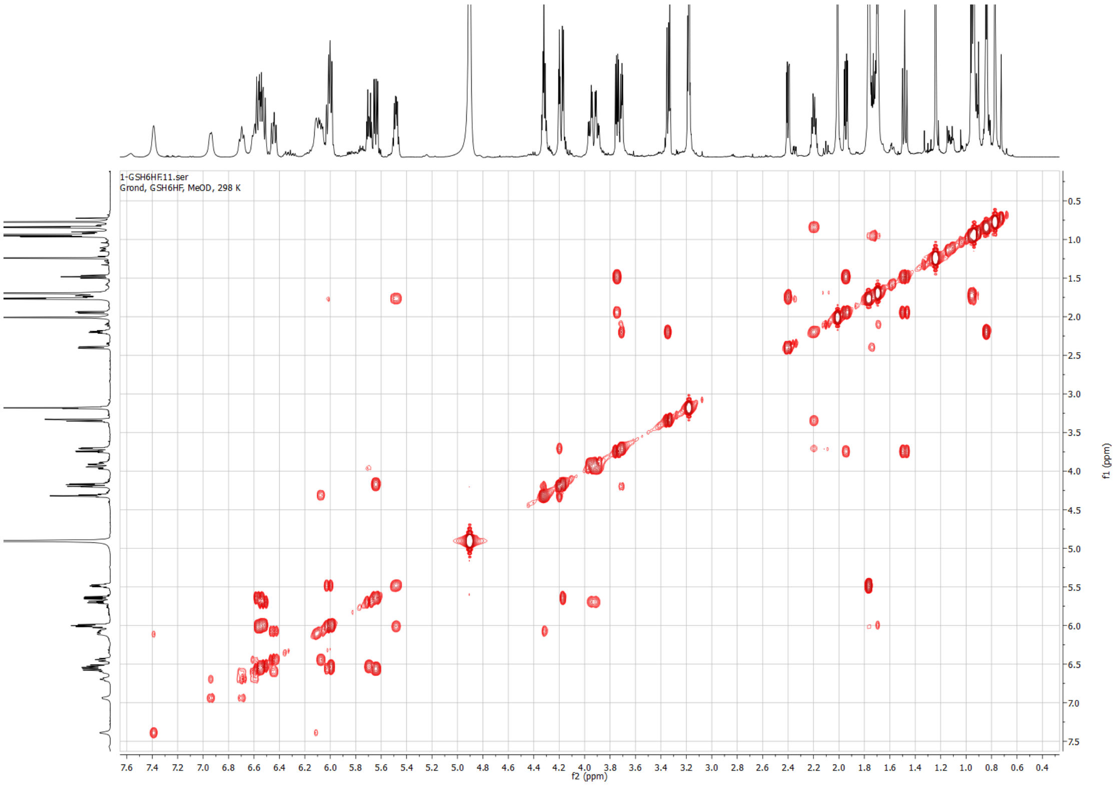

(IB) CH-correlation (HSQC) spectrum of 30-deoxy-kirromycin (3)

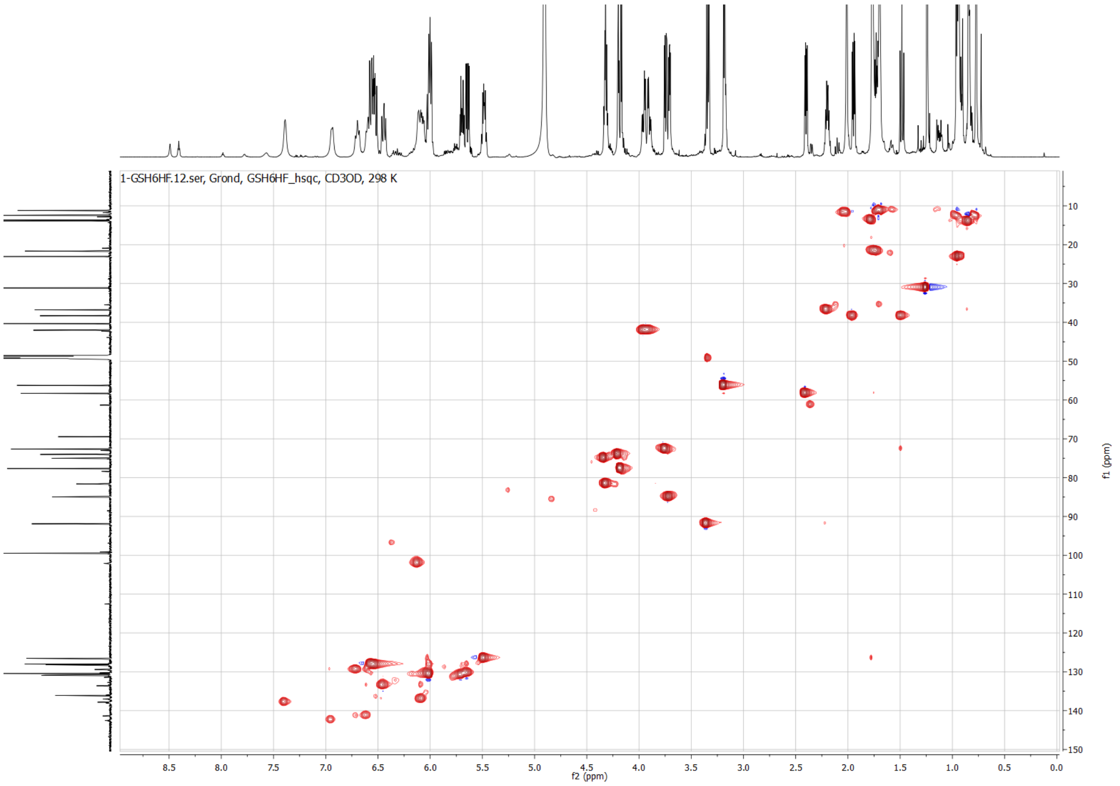

(IC) Multiple bond CH-correlation (HMBC) spec. of 30-deoxy-kirromycin (3)

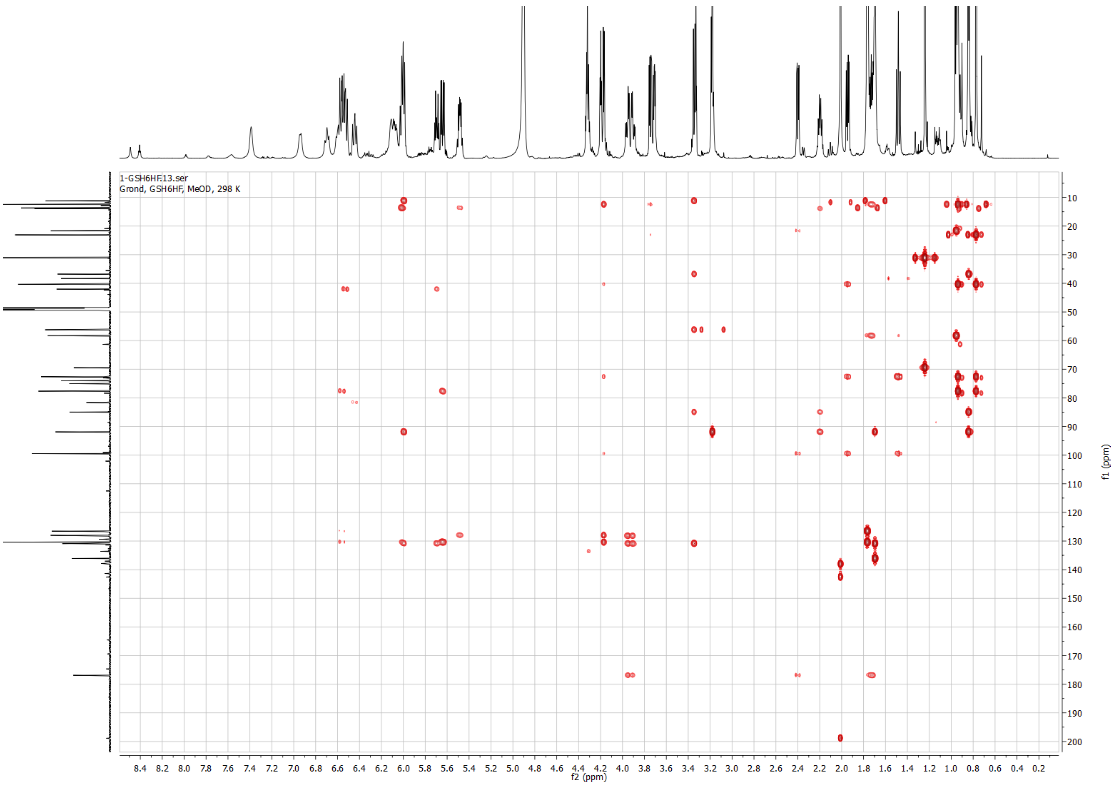

(ID) Key correlations highlighted for 30-deoxy-kirromycin (3)

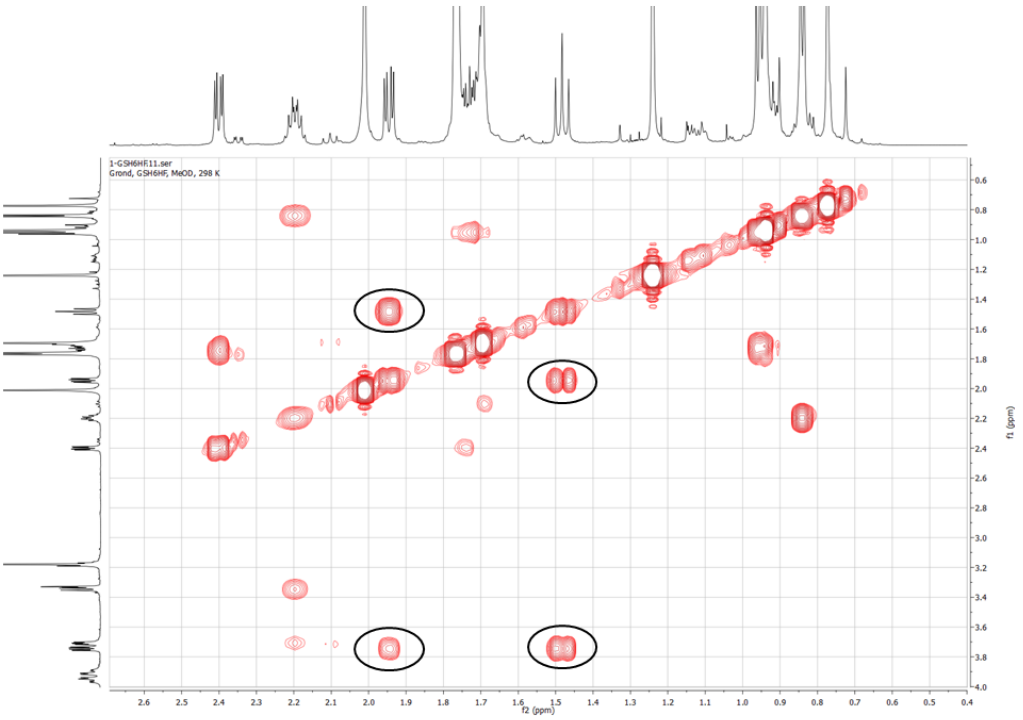

(II) 5,6-dihydro-kirromycin (4)

(IIA) In addition to the one-dimensional ^{1}H NMR spectra, the COSY spectrum gave evidence for the two additional methylene groups 5-H_2 (δ 2.54, t, broad) and 6-H_2 (δ 3.42, t, broad) with strong correlation signals (**IIA** and **IID**) for the 5,6-dihydro-kirromycin (**4**), isolated from the Δ*kirOI* mutant. **(IIB)** The HSQC gave evidence for the C-H connectivity. **(IIC)** The HMBC spectrum showed significant correlations of the intact kirromycin triene conjugated double bonds, e.g. 22-H (δ 5.96) with C-19 (δ_C 91.9) and C-43 (δ_C 11.2) and C-23 (δ_C 128.3) and, in addition, the correlation of 33-H (δ 4.21) with C-36 (δ_C 127.7).

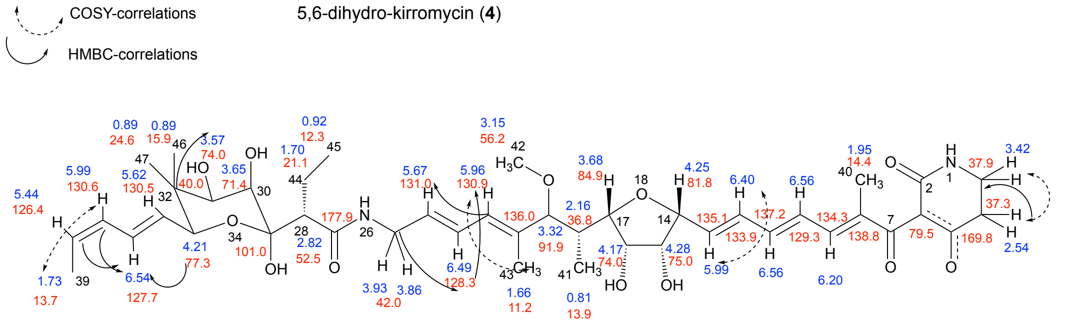

Formula S5-II Full assignment of ^{1}H and ^{13}C NMR data as example and selected COSY (double dashed arrowed) and HMBC (single arrowed) correlations for 5,6-dihydro-kirromycin (**4**).

(IIA) H-H-correlation (COSY) spectrum of 5,6-dihydro-kirromycin (4)

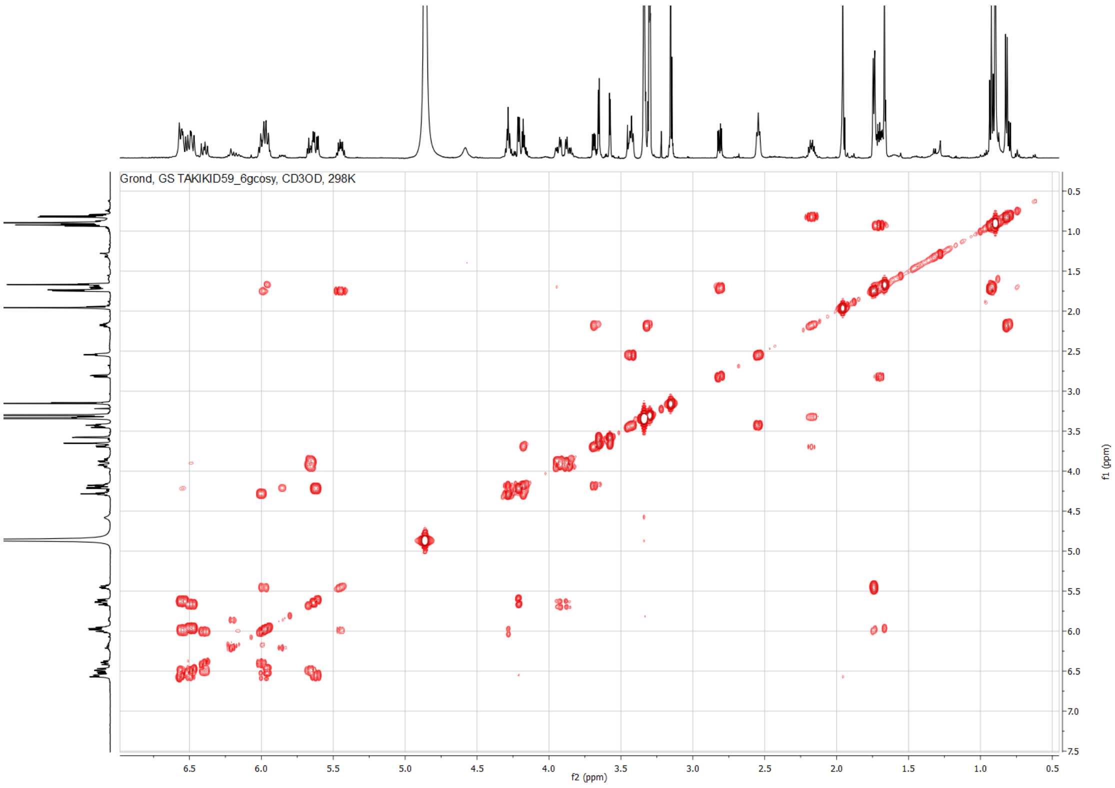

(IIB) CH-correlation (HSQC) spectrum of 5,6-dihydro-kirromycin (4)

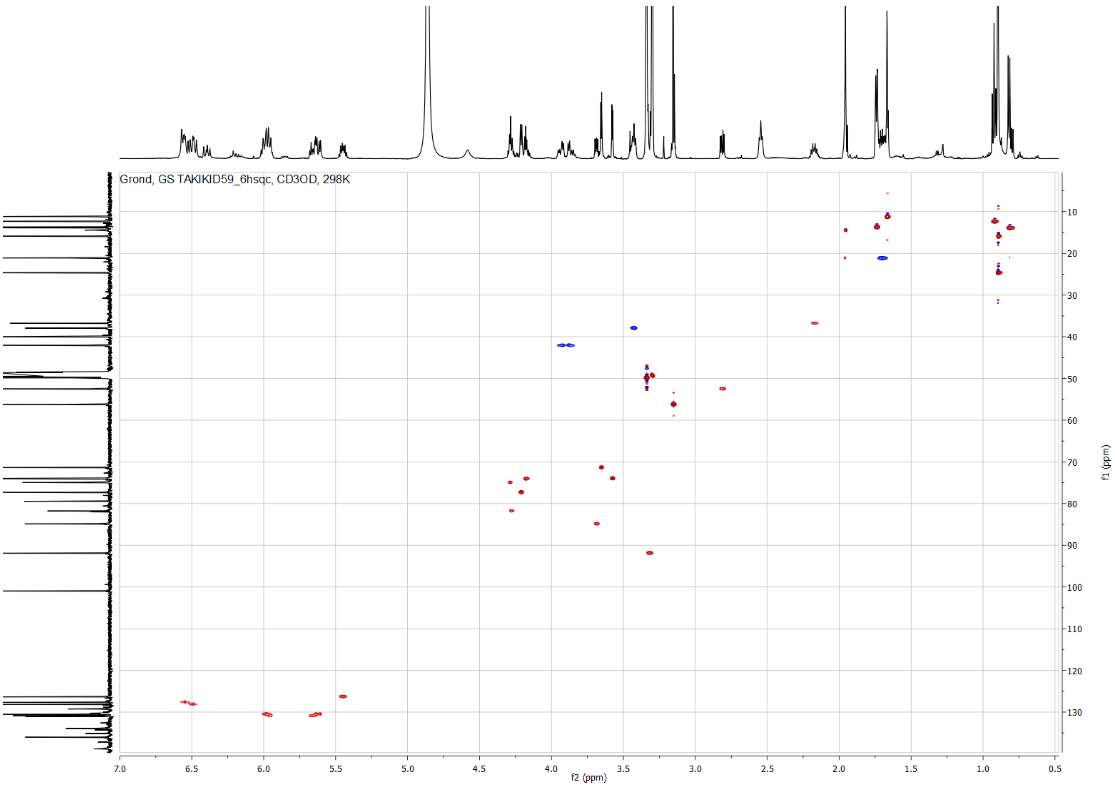

(IIC) Multiple bond CH-correlation (HMBC) spec. of 5,6-dihydro-kirromycin (4)

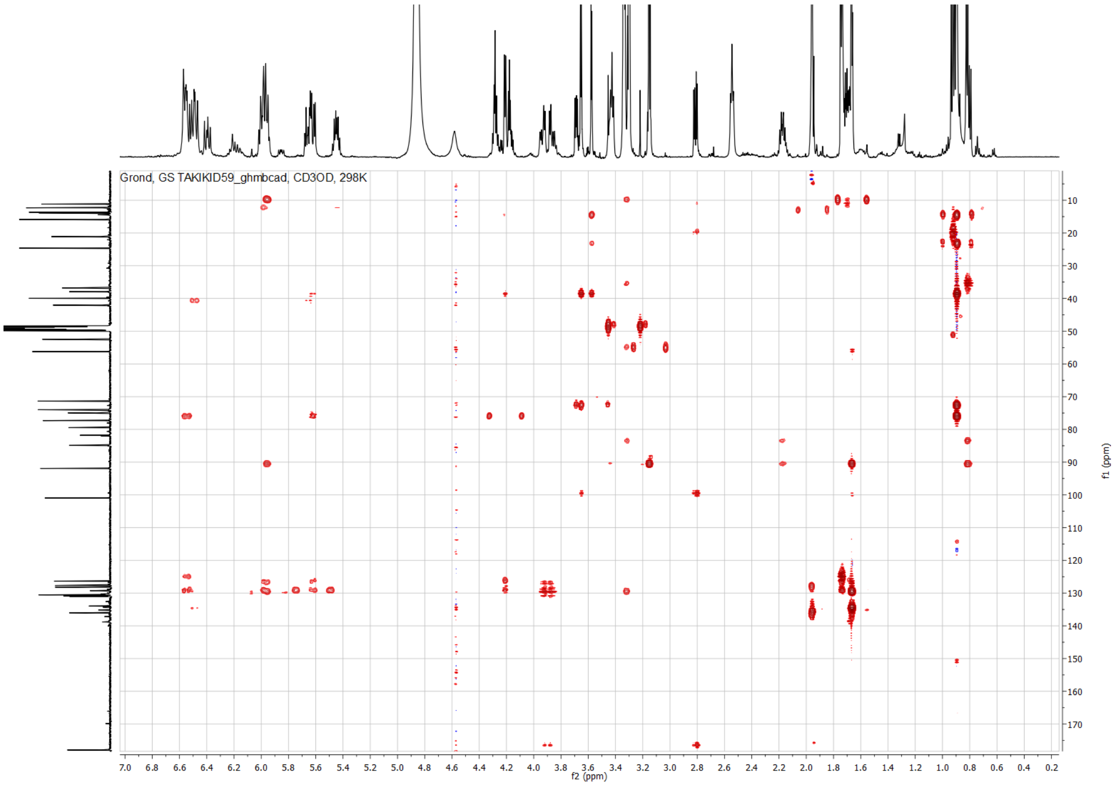

(IID) Key correlations highlighted for 5,6-dihydro-kirromycin (4)

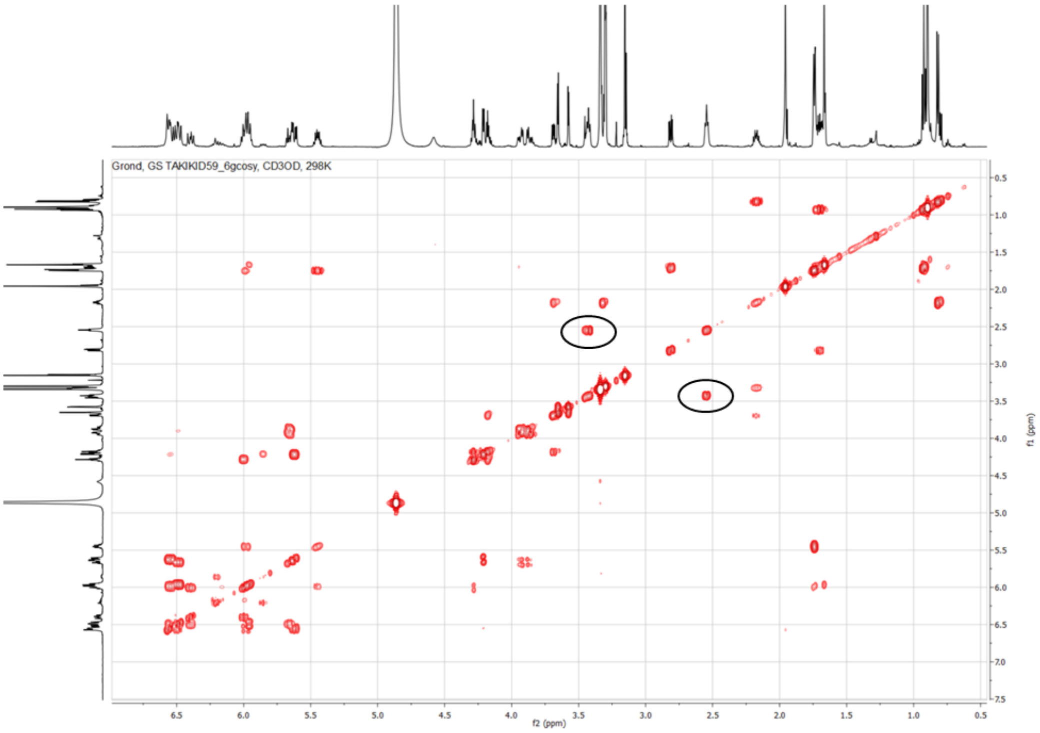

(III) 30-hydroxy-5,6-dehydro-1-*N*-demethyl-16-deoxy-kirrothricin (5)

COSY and Total Correlated Spectroscopy (TOCSY) spectra established the partial structure for the disrupted furan part (**IIIA**) in the 30-hydroxy-5,6-dehydro-1-*N*-demethyl-16-deoxy-kirrothricin (**5**) derivative isolated from the Δ*kirOII* mutant. H-17 (δ 4.06) exhibited correlations with both H-19 (δ 1.69) and H-16 (δ 2.40, 2.43, δ 2.25). H-16 exhibited further correlations to an olefinic proton H-15 (δ 5.90, 6.26), which confirmed the location of the extra double bond. **(II)** The HSQC gave evidence to the C-H connectivity. **(III)** The HMBC spectrum showed correlations between H-17 (δ 4.06), CH_3-41, C-16 (δ 40.0) and C-20 (δ 90.0), and corroborated the final structure of **5**. The new kirrothricin-like tetraene fragment was readily assigned from the HMBC-correlations of the CH-groups of C-9 to C-15 (see Formula **S5-III**). The red shifted UV-Vis spectrum for **5** could be explained by the elongation of the double bond chain.

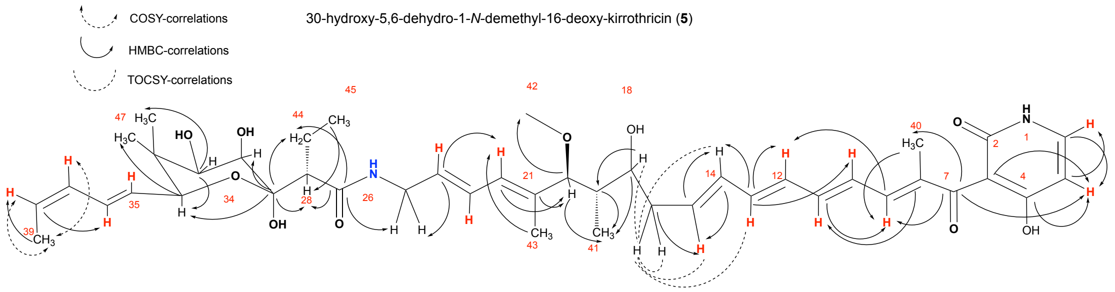

Formula S5-III Selected COSY (double dashed arrowed), TOCSY (dashed line), and HMBC (single arrowed) correlations for 30-hydroxy-5,6-dehydro-1-*N*-demethyl-16-deoxy-kirrothricin (**5**).

(IIIA) H-H-correlation (COSY) spectrum of 30-hydroxy-5,6-dehydro-1-*N*-demethyl-16-deoxy-kirrothricin (5)

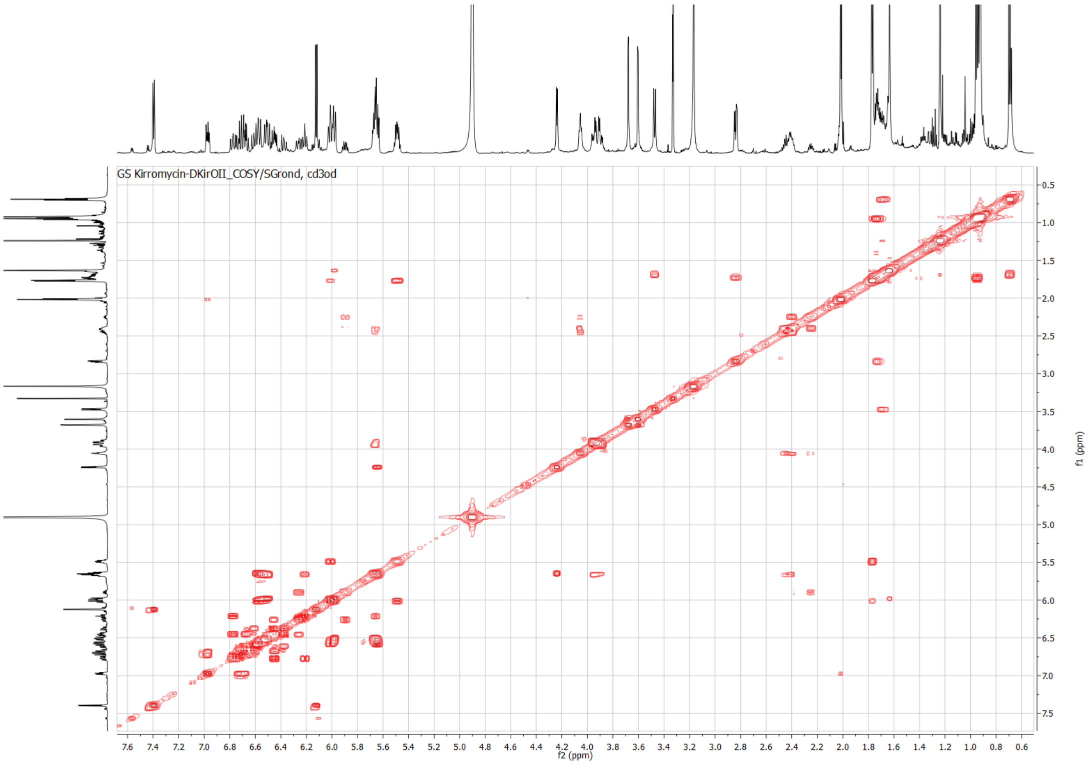

(IIIB) CH-correlation (HSQC) spectrum of 30-hydroxy-5,6-dehydro-1-*N*-demethyl-16-deoxy-kirrothricin (5)

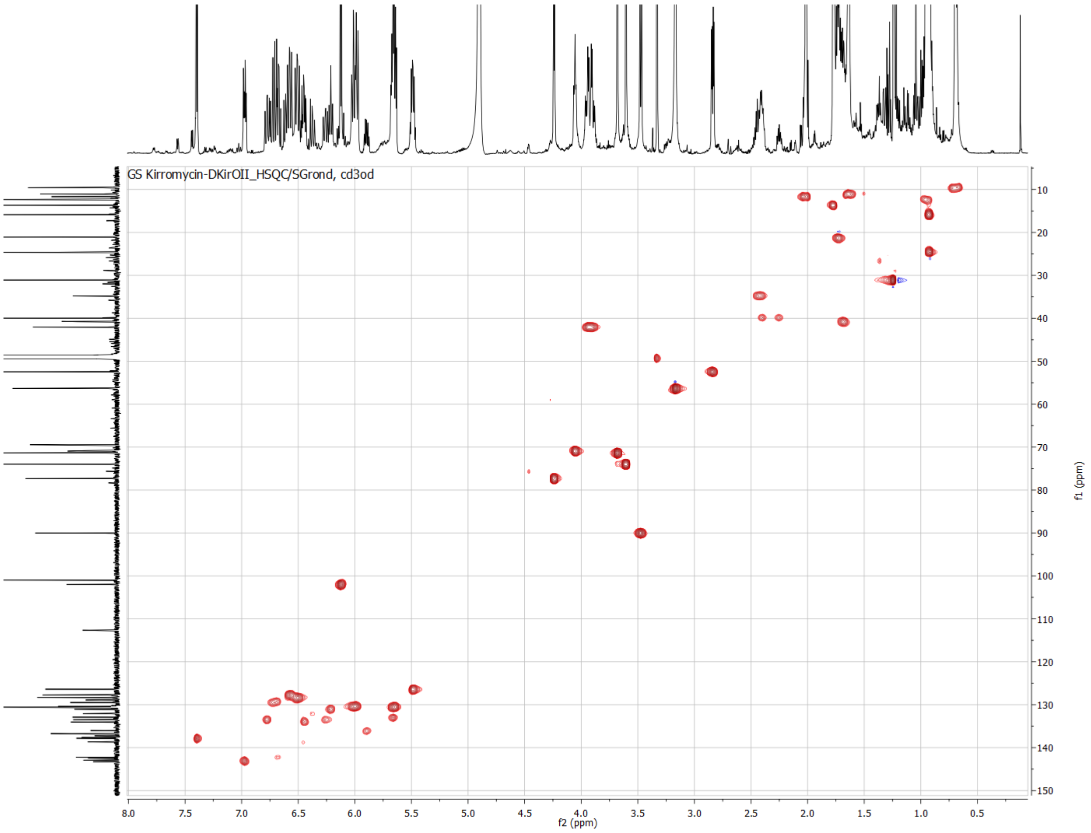

(IIIC) Multiple bond CH-correlation (HMBC) spec. of kirromycin 30-hydroxy-5,6-dehydro-1-*N*-demethyl-16-deoxy-kirrothricin (5)

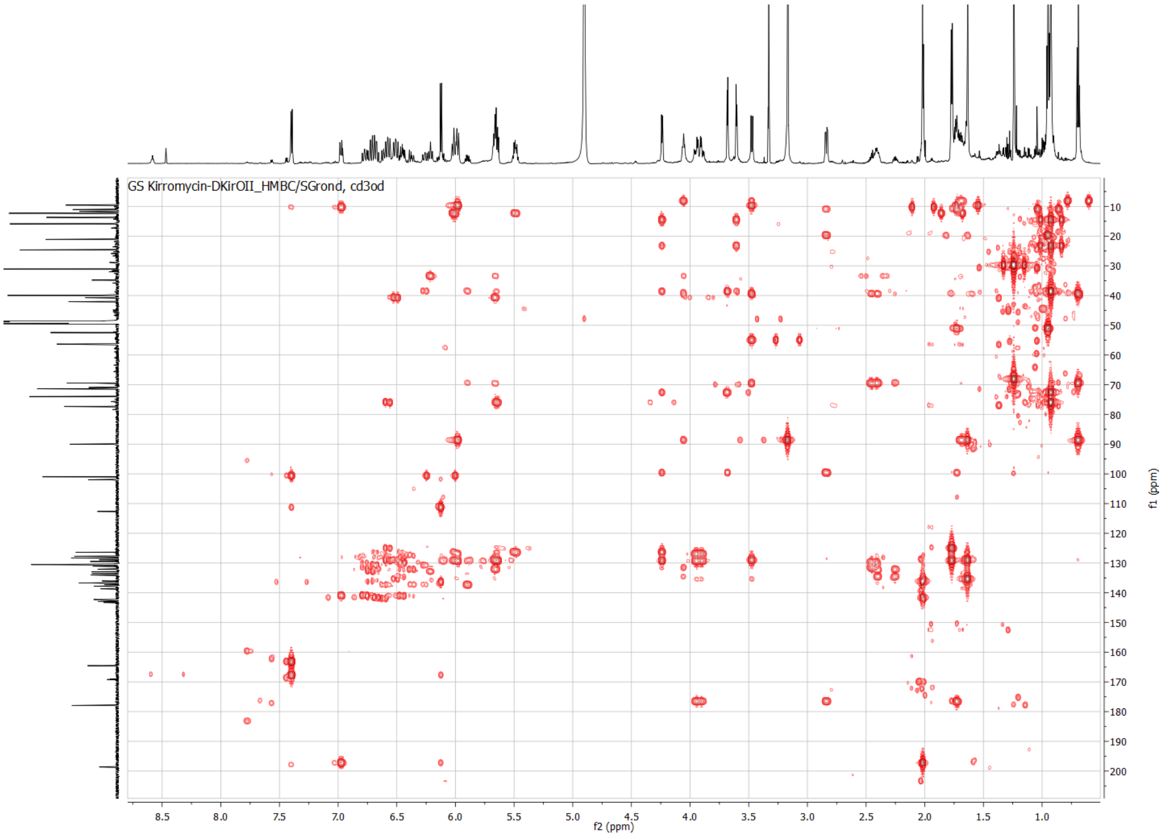

(IIID) Key correlations highlighted for 30-hydroxy-5,6-dehydro-1-*N*-demethyl-16-deoxy-kirrothricin (5)

HMBC-NMR experiment (CD_3OD, 700 MHz) showing key correlation of the new 16-CH_2-group (δ_H = 2.40, 2.43 ppm) with C-13, C-16, C-17 within the kirrothricin-type carbon chain of 30-hydroxy-5,6-dehydro-1-*N*-demethyl-16-deoxy-kirrothricin (**5**).

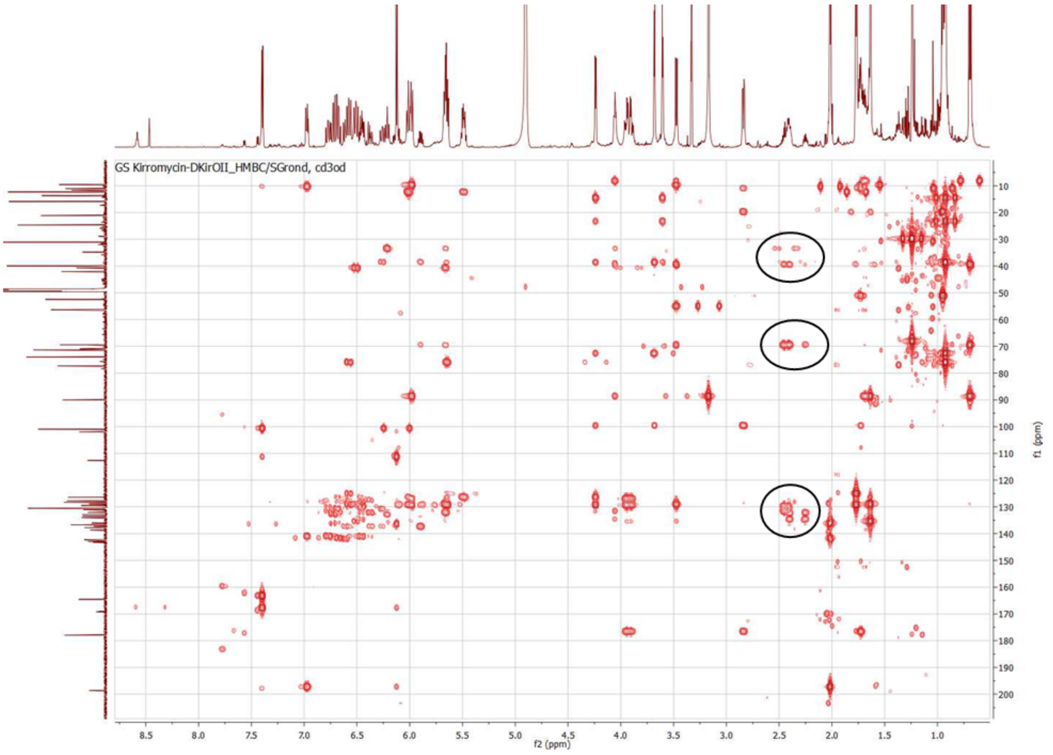

Figure S6-I – S6-II: Verification of Δ*kir* mutants by control PCRs

Control PCRs on genomic DNA (gDNA) of Δ*kirM,* Δ*kirHVI,* Δ*kirOI,* Δ*kirOII,* Δ*kirHIV,* Δ*kirHV,* and Δ*kirN* were carried out to confirm successful double crossover **(II)**. The use of primers (KP43 – KP56) targeting an internal 500 bp region of each gene in question **(I)** allowed for verification of correct mutants by the absence of bands (gene replaced by *ermE** promoter or thiostrepton resistance cassette). *Streptomyces collinus* Tü 365 (positive) (**1**) and water (negative) (**2**) controls were included for each primer pair. GeneRuler™ 1 kb DNA ladder (Fisher Scientific) (**L**) was used as marker.

(I)

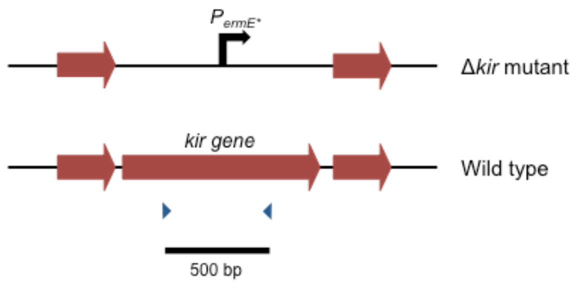

(II)

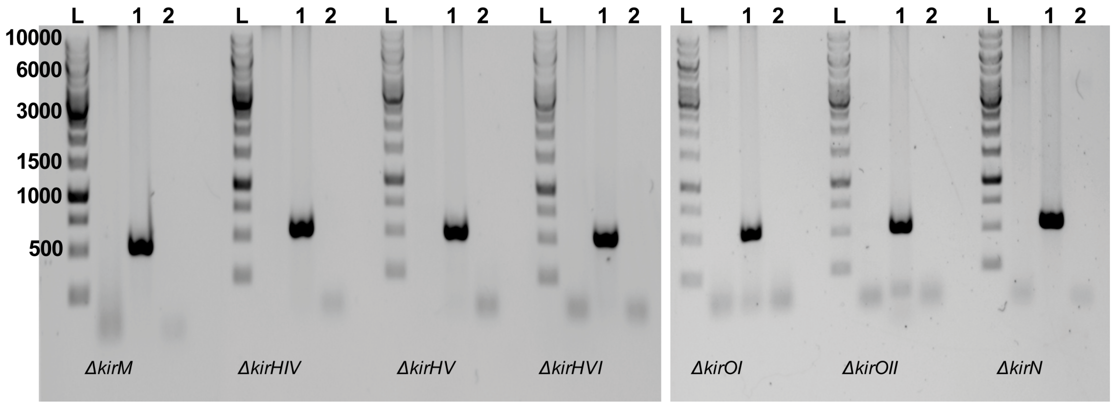

Figure S7-I – S7-III: Putative enzymatic reactions catalysed by tailoring enzymes KirHVI, KirOI, and KirOII

Genetic inactivation studies and NMR analysis enabled the prediction of the putative enzymatic reactions catalysed by the phytanoyl-CoA dioxygenase KirHVI (encoded by *kirHVI*) **(I)** and the two P450-dependent hydroxylases KirOI and KirOII (encoded by *kirOI* **(II)** and *kirOII* **(III)**).

(I)

Based on its similarity to the hydroxylase Fum3p involved in fumonisin B biosynthesis[10], KirHVI is postulated to induce α-ketoglutarate (α-KG)/Fe(II)-dependent hydroxylation at the C-30 position in the sugar-like moiety in kirromycin.

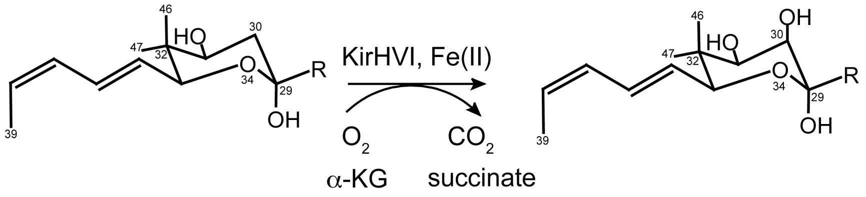

(II)

The cytochrome P450-dependent hydroxylase KirOI catalyses hydroxylation of either C-5 or C-6 in the δ-lactam ring of the kirromycin biosynthetic intermediate (i). The hydroxylated product from the Δ*kirOI* mutant was not be detected in either the culture broth or the solvent extract. The intermediate can then undergo dehydration, which might be facilitated by a yet uncharacterised enzyme or an in *trans*-acting dehydrogenase (DH), to form the double bond between C-5 and C-6 (ii). The product **ii** bears the stable pyridone ring with tautomeric equilibrium and aromatic character, presumably responsible for the stability of this dehydrated product.

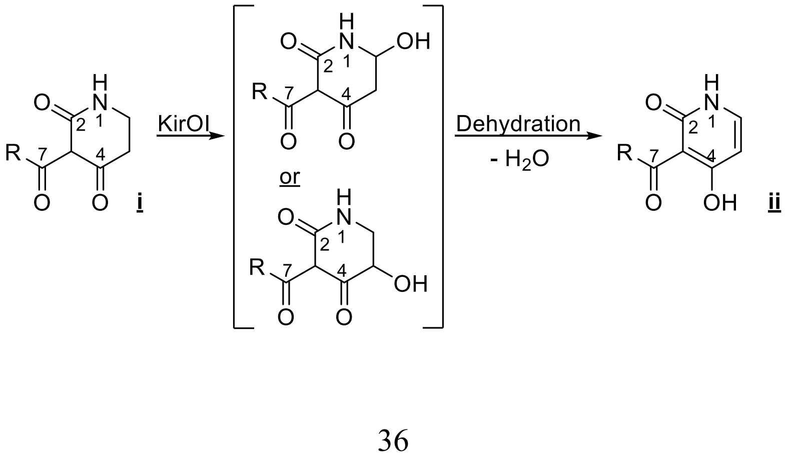

(III)

Our data suggests KirOII to be involved in formation of the tetrahydrofuran (THF) ring in the centre of the kirromycin molecule.

In case of kirromycin biosynthesis, the PKS-derived precursor molecule of kirromycin could undergo dual CH_2-hydroxylation at C-14 and C-16, possibly catalysed by the cytochrome P450 monooxygenase KirOII. The hypothesis is strengthened from the sequence comparison of KirOII to AurH (31 % identity and 49 % similarity). The cytochrome P450 monooxygenase AurH has been found to be involved in formation of the THF ring in the antifungal compound aureothin produced by *Streptomyces thioluteus*[11,12] through the introduction of two C-O bonds. In a similar fashion, KirOII might catalyse the following step, including a classical ring-closing dehydration reaction under formation of the THF ring moiety in kirromycin. This conversion is favoured in kirromycin because of increased nucleophilicity of the C-17-hydroxyl group as a result of the hydrogen bonding in a cyclic 6-membered ring including the C-20 carbonyl group. From the Δ*kirOII* mutant, the tetraene product, named 30-hydroxy-5,6-dehydro-1-*N*-demethyl-16-deoxy-kirrothricin (**5**), is isolated from the culture broth due to chemically favoured dehydration to the stable extended conjugated double bond system between C-6 and C-15.

While this is a plausible enzymatic reaction, the scheme currently lacks an additional enzyme responsible for catalysing the reduction of C=O to C-OH at the C-20 position in kirromycin. Such an enzyme remains to be identified and characterized.

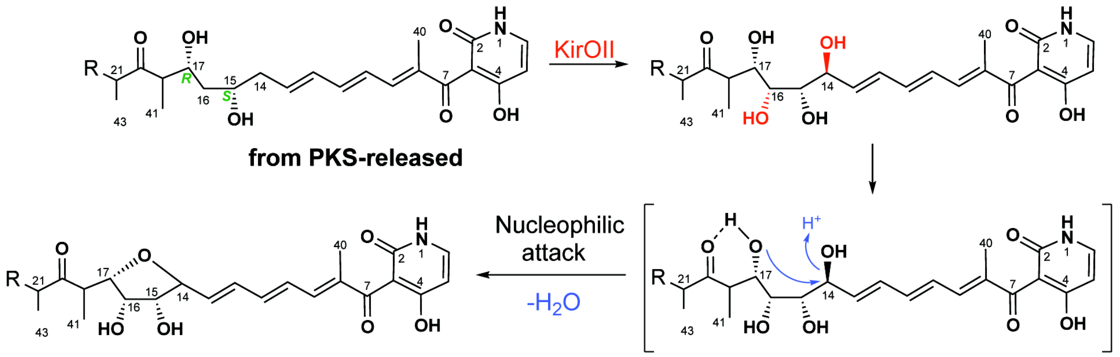

Supplementary references

1. MacNeil, D. J. *et al.* Analysis of *Streptomyces avermitilis* genes required for avermectin biosynthesis utilizing a novel integration vector. *Gene* **111,** 61–68 (1992).
2. Wolf, H. & Zähner, H. Stoffwechselprodukte von Mikroorganismen. *Arch. Mikrobiol.* **83,** 147–154 (1972).
3. Weber, T. *et al.* Molecular Analysis of the Kirromycin Biosynthetic Gene Cluster Revealed β-Alanine as Precursor of the Pyridone Moiety. *Chem. Biol.* **15,** 175–188 (2008).
4. Myronovskyi, M., Welle, E., Fedorenko, V. & Luzhetskyy, A. Beta-glucuronidase as a sensitive and versatile reporter in actinomycetes. *Appl. Environ. Microbiol.* **77,** 5370–5383 (2011).
5. Menges, R., Muth, G., Wohlleben, W. & Stegmann, E. The ABC transporter Tba of *Amycolatopsis balhimycina* is required for efficient export of the glycopeptide antibiotic balhimycin. *Appl. Microbiol. Biotechnol.* **77,** 125–134 (2007).
6. Muth, G., Wohlleben, W. & Puhler, A. The minimal replicon of the *Streptomyces ghanaensis* plasmid pSG5 identified by subcloning and Tn5 mutagenesis. *Mol. Gen. Genet.* **211,** 424–429 (1988).
7. Tong, Y., Charusanti, P., Zhang, L., Weber, T. & Lee, S. Y. CRISPR-Cas9 Based Engineering of Actinomycetal Genomes. *ACS Synth. Biol.* **4,** 1020–1029 (2015).
8. Tong, Y., Robertsen, H. L., Blin, K., Weber, T. & Lee, S. Y. in *Methods in Molecular Biology* **1671,** 163–184 (2018).
9. Frost, B. M., Valiant, M. E. & Dulaney, E. L. Antibacterial Activity of Heneicomycin. *J. Antibiot*. **32**, 626–629 (1979).
10. Ding, Y., Bojja, R. S. & Du, L. Fum3p, a 2-Ketoglutarate-Dependent Dioxygenase Required for C-5 Hydroxylation of Fumonisins in *Fusarium verticillioides*. *Appl. Environ. Microbiol.* **70,** 1931–1934 (2004).
11. He, J. & Hertweck, C. Iteration as Programmed Event during Polyketide Assembly; Molecular Analysis of the Aureothin Biosynthesis Gene Cluster. *Chem. Biol.* **10,** 1225–1232 (2003).
12. Richter, M. E. A., Traitcheva, N., Knüpfer, U. & Hertweck, C. Sequential Asymmetric Polyketide Heterocyclization Catalyzed by a Single Cytochrome P450 Monooxygenase (AurH). *Angew. Chemie* **120,** 9004–9007 (2008).
